# Plate-based 10X Genomics-compatible single-cell RNA-sequencing based on Smart-seq3xpress

**DOI:** 10.1186/s12864-025-12286-2

**Published:** 2025-12-06

**Authors:** Koen Deserranno, Elise Callens, Danique Berrevoet, Aaron Verplancke, Tamara De Vos, Tom Taghon, Dieter Deforce, Filip Van Nieuwerburgh

**Affiliations:** 1 Laboratory of Pharmaceutical Biotechnology – NXTGNT, Faculty of Pharmaceutical Sciences, Ghent University, Ottergemsesteenweg 460, BLOK A, FLOOR3, Ghent, 9000 Belgium; 2 Department of Diagnostic Sciences, Ghent University, Ghent, Belgium

**Keywords:** Plate-based scRNA-seq, 10X Genomics, Smart-seq, Indexed sorting, Benchmarking

## Abstract

**Background:**

10X Genomics (10X) is a leading single-cell RNA-sequencing (scRNA-seq) technology that permits gene expression and T-cell receptor (TCR) repertoire profiling. However, its microfluidics-based approach restricts direct pairing of upstream indexed single-cell sorting with downstream scRNA-seq data and poses challenges for cost-effective profiling of small-scale cell populations.

**Results:**

To address these limitations, we developed a plate-based, 10X-compatible (PB10X) scRNA-seq strategy built on the Smart-seq3xpress (SS3X) principle. PB10X supports indexed sorting using Fluorescence Activated Cell Sorting (FACS) directly into 384-well plates and generates cDNA compatible with any standardized 10X Single Cell 5’ library construction kit including the 5’ V(D)J and 5’ Gene Expression kits. PB10X offers the flexibility of plate-based scRNA-seq with the robustness of the 10X sequencing library preparation, while retaining full compatibility with downstream Cell Ranger data processing.

We demonstrated PB10X’s performance on Jurkat lymphoblasts by generating V(D)J and gene expression sequencing libraries, and benchmarking these against 10X and SS3X. PB10X proved particularly effective for TCR repertoire sequencing, yielding paired TCRαβ chains for 81.71% of the cells at a limited sequencing depth. PB10X further detected a mean of 4,343 genes and 16,137 UMIs per cell, while achieving the highest proportion of uniquely mapped reads and protein-coding genes among the compared methods. Notably, PB10X resolves the strand invasion artifact observed in SS3X.

**Conclusions:**

The novel cost-effective PB10X method offers unprecedented 10X-compatibility in a plate-based format, both for immune receptor repertoire sequencing and gene expression profiling, representing a promising single-cell transcriptomics strategy, especially in immunological settings with limited cell populations.

**Supplementary Information:**

The online version contains supplementary material available at 10.1186/s12864-025-12286-2.

## Background

Various single-cell RNA-sequencing (scRNA-seq) strategies have been developed in the last decade, enabling the observation of rare cells and the heterogeneity within cell populations. While all single-cell sequencing strategies share core steps—cell lysis, reverse transcription, barcoding, cDNA amplification, library construction, and sequencing—they differ in critical aspects. These variations significantly impact experimental design, cell input requirements, data richness and complexity, sensitivity, cost-effectiveness, throughput capacity, and analytical approaches. Each method's unique combination of these factors influences its suitability for specific research questions and experimental designs [1, 2]. Key differences among single-cell sequencing methods include the single-cell isolation strategy (droplet-based, microwell-based, plate-based), the cell input requirements (ability to work with fixed cells, necessity for live cell input) and transcript coverage (full-length transcript sequencing vs partial 3' or 5' end transcript sequencing) [3, 4]. In this study, we focus on these differences between the droplet-based 10X Genomics (10X) method and the plate-based “Switching Mechanism at the 5′ end of RNA Template-sequencing” (SMART-seq) method [5–7]. To capitalize on the strengths of both approaches, we provide a novel plate-based, SMART-seq-inspired method (PB10X) that generates barcoded cDNA that is compatible with any commercially available 10X Single Cell 5’ library construction kit.

The 10X Chromium system stands out as the most widely used droplet-based scRNA-seq platform [5]. The system utilizes microfluidic technology to partition single cells into Gel Beads-in-emulsion (GEMs), enabling the parallel processing of thousands of cells from a single sample. Despite being limited to analyze only the 3' or 5' ends of cDNA using short-read sequencing protocols, the 10X platform provides extensive analysis possibilities. These include assessing T-cell receptor (TCR) and B-cell receptor (BCR) sequences, cell surface protein expression, and gene expression from the same cDNA through different library construction kits. However, due to its reliance on microfluidic technology and relatively lower cell capture efficiency, performing 10X on small cell populations is challenging, and results in a high cost per cell for these limited cell numbers [8, 9]. This can be a limiting factor in clinical settings where large cell quantities are not available [10]. BD Rhapsody, another scRNA-seq platform offering similar capabilities, faces the same limitation in that at least ≥ 1,000 cells must be loaded, and a typical cell recovery rate of only 50–65% [11, 12].

In contrast, plate-based SMART-seq strategies boast full-transcript coverage and increased sensitivity [13]. Despite its inherent lower throughput, mostly limited to 384-well plate format, the latest SMART-seq iteration, Smart-seq3xpress (SS3X), is automatable and can be harnessed to analyze a larger number of cells [13]. Similar to the 10X library preparation, SS3X generates full-length cDNA using oligo-dT priming and template-switching. However, unlike 10X, where the full-length cDNA is fragmented and only the transcript ends are amplified, SS3X achieves full-length transcript coverage by tagmentation of the cDNA and subsequently amplifying both the 5' and internal fragments [13]. Additionally, SS3X offers a unique feature for partial transcript and isoform reconstruction using short-read sequencing. This is achieved by *in silico* linking of 5’ end fragments that share the same unique molecular identifier (UMI) sequence but differ in their 3’ transposase insertion sites [13, 14]. While both droplet- and plate-based strategies permit prior Fluorescence Activated Cell Sorting (FACS) of the cell population of interest, only plate-based strategies allow direct pairing of the phenotypical cell characteristics with the transcriptome data of the same cell through ‘indexed’ sorting [8].

Beyond differential gene expression, the field of scRNA-seq has expanded to include techniques for TCR and BCR repertoire sequencing. TCR and BCR repertoire sequences can be *in silico* reconstructed directly from the SS3X data using dedicated algorithms such as TRUST4, TraCeR, or BraCeR [15–17]. For 10X, the preparation and sequencing of two independent libraries, i.e. gene expression and amplicon-based V(D)J enrichment, is required to obtain the same information that is retrieved in a single sequencing run for SS3X.

Here, we present a novel plate-based 10X-compatible (PB10X) scRNA-seq strategy that allows for cDNA-generation compatible with any downstream commercially available 10X Single Cell 5’ library construction kit, and supports single-cell indexed sorting directly in a 384-well plate. Compared to droplet-based 10X, which requires a relatively high number of input cells, PB10X can be performed on only a few cells. Compared to SS3X, this approach introduces the cell-specific barcode in the Template Switching Oligonucleotide (TSO), enabling pooling of the cDNA of all samples earlier in the protocol. This allows all subsequent protocol steps to be carried out in a single reaction tube, significantly reducing labor-intensive manual handling. Furthermore, PB10X allows for an amplicon-based enrichment of B- and T-cell receptor sequences with commercially available 10X Genomics kits, allowing a lower sequencing depth per cell while providing enhanced immune repertoire data. We applied PB10X to Jurkat T lymphoblasts and validated its compatibility with both the 10X Single Cell 5’ Gene Expression as 5’ V(D)J library construction from the same 5′ 10X-compatible cDNA. We benchmarked the PB10X method to 10X 5’ and SS3X for the same Jurkat cell type in terms of library preparation, scRNA-seq quality metrics, sequencing depth, gene dropouts, gene expression, and TCR sequence reconstruction capabilities.

## Results

### Plate-based 10X workflow

**Fig. 1:**
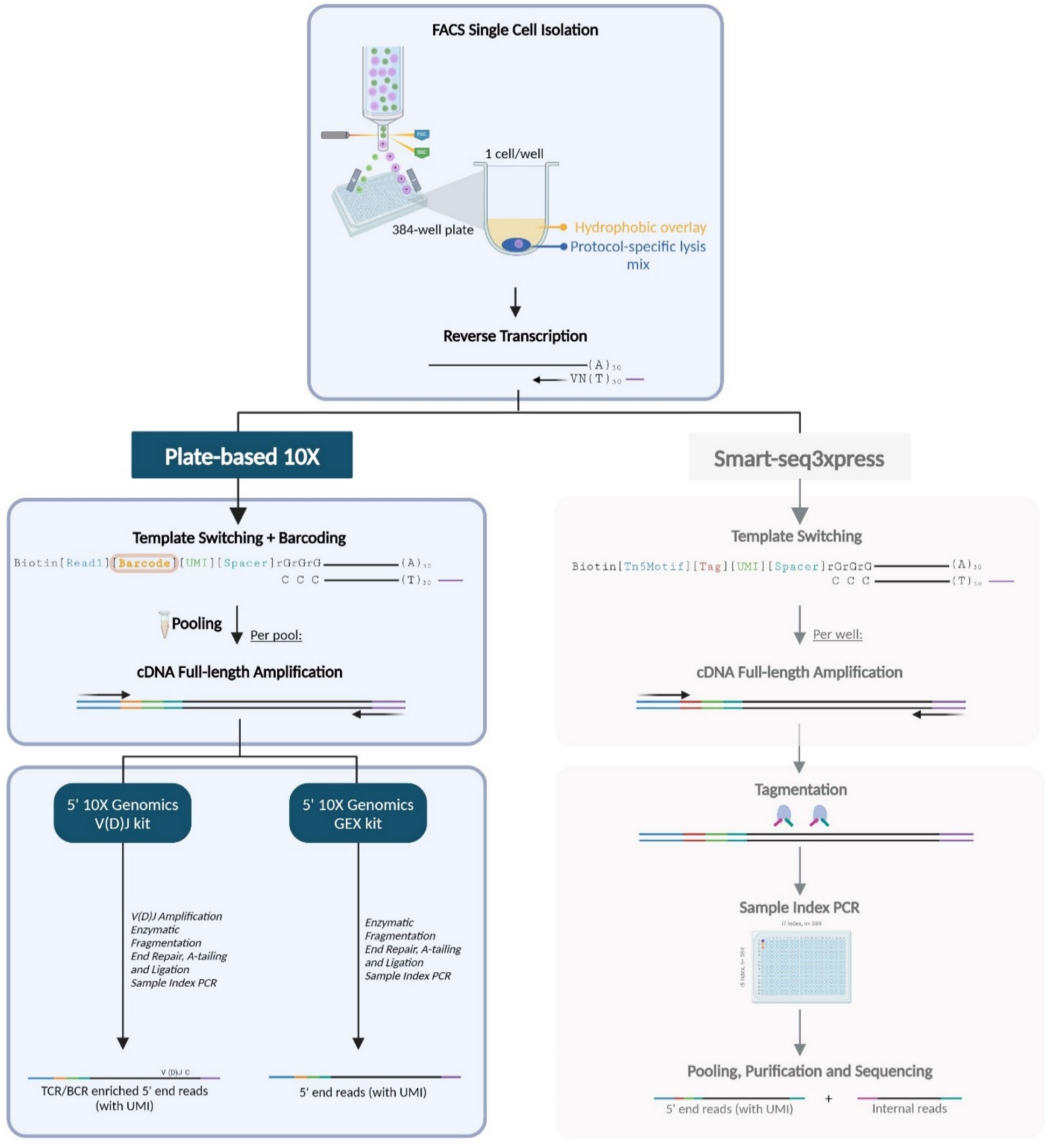
Schematic outline of the PB10X method in comparison with SS3X. (Left) Library strategy for PB10X. Released PolyA+ RNA molecules originating from FACS-sorted cells are reverse transcribed, and template switching is carried out at the 5′ end using a custom barcoded 10X-compatible TSO. After pooling and cDNA pre-amplification any commercially available 10X Single Cell 5’ library construction kit can be used for downstream processing. (Right) Library strategy for SS3X. Released PolyA+ RNA molecules originating from FACS-sorted cells are reverse transcribed, and template switching is carried out at the 5′ end using the regular unbarcoded SS3X TSO. After PCR pre-amplification, tagmentation introduces near-random cuts in the cDNA, producing 5′ UMI-tagged fragments and internal fragments spanning the whole gene body [18]

Our PB10X assay (Fig. 1, left) is based on the design of the existing plate-based and miniaturized SS3X protocol (Fig. 1, right). The primary innovation of the assay lies in the use of custom 10X-compatible TSOs that incorporate a cell-specific barcode into the TSO (Supplementary Materials Table S1). This feature enables downstream processing with a single pooled reaction instead of a reaction per individual well in SS3X, rendering a time-efficient protocol. Furthermore, it ensures that the library is compatible with any commercially available downstream standardized 10X Single Cell 5’ library construction kit such as the 10X Single Cell 5’ Gene Expression and V(D)J library construction kits. It is important to note that, unlike SS3X, PB10X does not generate full-length sequencing libraries. Although full-length cDNA is synthesized during reverse transcription and amplified within the cDNA pre-amplification step, downstream use of the 10X 5′ library preparation kits includes a fragmentation step, thereby precluding true full-length transcript coverage.

With PB10X, single cells of interest are sorted into individual wells of a 384-well plate using FACS. Single cells are collected into a prefilled 384-well plate containing the nanoliter-sized SS3X lysis mix. Notably, compared to the regular SS3X protocol, a custom barcoded 10X-compatible TSO is added to each well at this stage. Our final TSO design is based on the barcoded 10X TSO design and includes a 5’ biotin-group, the 10X PCR-handle read 1 sequence, a 16-bp 10X well-specific barcode followed by a 9-bp UMI, the 10X TTTCTTATAT spacer, and three riboguanines. When designing our TSO, the use of 5’-biotin-blocked TSOs was compared to 5’-unblocked TSOs. Using an unblocked 5’-end TSO led to a hedgehog pattern in the Fragment Analyzer electropherograms of pre-amplified cDNA libraries (Supplementary Materials Fig. S1). Long-read Oxford Nanopore Technologies (ONT) sequencing of the cDNA confirmed the presence of TSO concatemer sequences (Supplementary Materials Fig. S2). While TSO concatemerization, caused by secondary template switching reactions at the 5’-end, is a well-documented phenomenon, it is often overlooked in practical protocol implementations. This reaction is mainly favored in small RNA quantities such as found in T-cells [19–22]. The presence of TSO concatemerization in standard short-read Illumina sequencing data resulted in roughly 60% of the reads being concatemers, dramatically reducing the percentage of uniquely mapped reads to only 2%. Although 5’-end blocking of the TSO is not a novel strategy, it was a necessary adaptation for our 10X-inspired TSOs, where normally the presence of the 10X gel bead prevents concatemerization. We finally chose to block the 5’-OH group of the TSO with a biotin group, resulting in a significant improvement of library complexity with complete elimination of these problematic concatemers. Finally, 24 well-specific barcodes were selected from the 10X barcode whitelist, each differing with a minimal Levenshtein distance of 5 between any two barcodes. For large-scale experiments, additional custom barcoded TSOs could be developed to enable downstream processing with only a single pool.

### Efficient TCR reconstruction with PB10X-V(D)J at limited sequencing depth

**Fig. 2:**
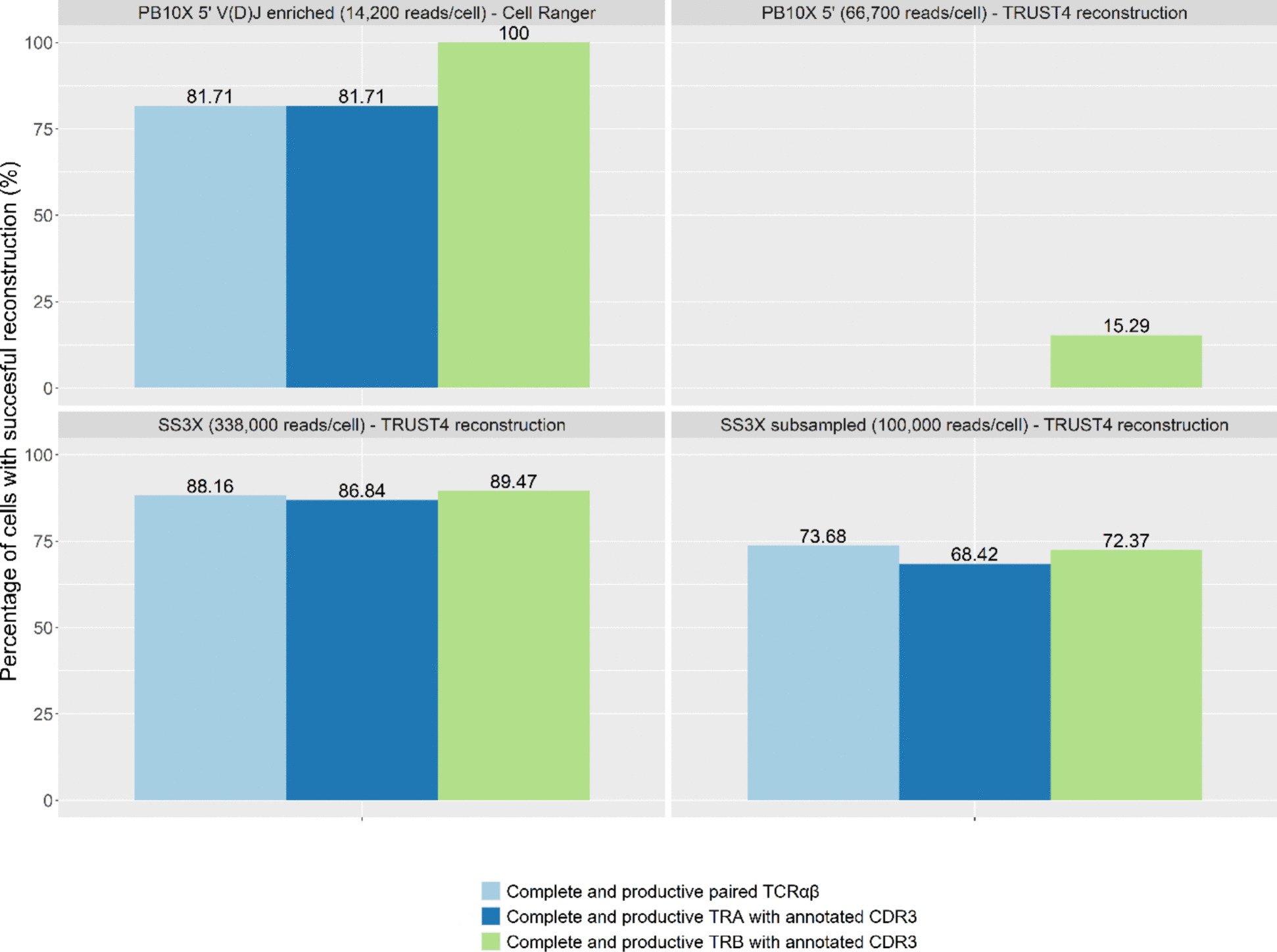
Percentages of cells with successful TCR reconstruction across the different strategies. The percentage of cells with successful reconstruction of a complete, productive TCR α-chain, TCR β-chain and TCR αβ-pair are displayed. For analysis of TCR recovery rates of the PB10X-V(D)J-enriched strategy Cell Ranger was used, while for TCR sequence reconstructions based on SS3X and PB10X 5’ without specific V(D)J enrichment the TRUST4 algorithm was used

We assessed the performance of PB10X in conjunction with the downstream amplicon-based 10X Single Cell 5’ V(D)J library construction kit, hereafter referred to as PB10X 5’ V(D)J-enriched, starting from the same cDNA as used for gene expression profiling before. The Jurkat T lymphoblast line, known for its well-characterized TCR (TRAV8-4-CAVSDLEPNSSASKIIF-TRAJ3; TRBV12-3-CASSFSTCSANYGYTF-TRBJ1-2, Clone E6-1), served as a reference point to assess the effectiveness of the different library preparation strategies in retrieving the TCR sequence [23, 24]. We compared the PB10X TCR amplicon sequences to TRUST4 TCR sequence reconstructions from SS3X, in terms of successful reconstruction for the TCR alpha and beta locus, both individually as their paired occurrence, as illustrated in Fig. 2. The Jurkat-specific clonotype was confirmed in both the PB10X and SS3X datasets.

PB10X 5’ V(D)J-enriched achieved 81.71% recovery of complete, productive, and CDR3-annotated TCR pairs at a targeted sequencing depth of only ~ 14,200 reads per cell. To note, one pool of the PB10X V(D)J cDNA libraries deviated from the typical prominent V(D)J peak, normally observed during capillary electrophoresis-mediated quality control of the final sequencing library, and displayed an overall shorter fragment length distribution, despite parallel processing. Consequently, this individual pool yielded fewer cells with CDR3-annotated TRA contigs, possibly lowering the overall TCR sequence recovery rate of the PB10X 5’ V(D)J enriched strategy. The SS3X data (~ 338,000 reads/cell) illustrated similar TCR recovery rates based on the TRUST4 reconstruction without prior enrichment. BD recently reported that using their Rhapsody system in combination with TCR/BCR Next Multiomic Assays (WTA + VDJ + Abseq) on human PBMCs, 86% of TCR alpha and beta pairing efficiency was obtained [25]. These results are similar compared to our TCR pairing efficiencies for Jurkat cells obtained using PB10X 5’ VDJ and SS3X, however without the need for a dedicated microwell-based instrument.

When the SS3X data, including both 5’ UMI and internal reads, was subsampled to 100,000 reads per cell, which is generally considered the minimal depth of the SS3X approach, about 73.68% of the TCR pairs could be reconstructed. To further assess the impact of sequencing depth on reconstruction success rate, we subsampled the SS3X dataset to equal sequencing depth of the PB10X V(D)J enriched dataset (14,200 reads/cell). The paired TCR reconstruction success rate of SS3X dropped drastically to 1.32% (Supplementary Materials Fig. S3). As expected, the main limitation for reconstruction was the shallow sequencing depth.

Subsequently, we investigated if subsampling of the PB10X V(D)J dataset to 1,666 reads/cell, i.e. the mean number of reads per cell aligned to the *TRA* or *TRB* genes in the complete SS3X dataset, resulted in similar TCR profiling performance (Supplementary Materials Fig. S3C). In these circumstances, SS3X retrieves a higher percentage of complete and productive paired TCRαβ than the PB10X V(D)J dataset. However, it is of importance to emphasize that to achieve these results and the mean number of 1,666 SS3X reads per cell aligning to the *TRA* or *TRB* gene, very deep sequencing to 338,000 overall SS3X reads was required, as no direct options are available to just sequence the SS3X reads corresponding to the *TRA* or *TRB* genes.

**Fig. 3:**
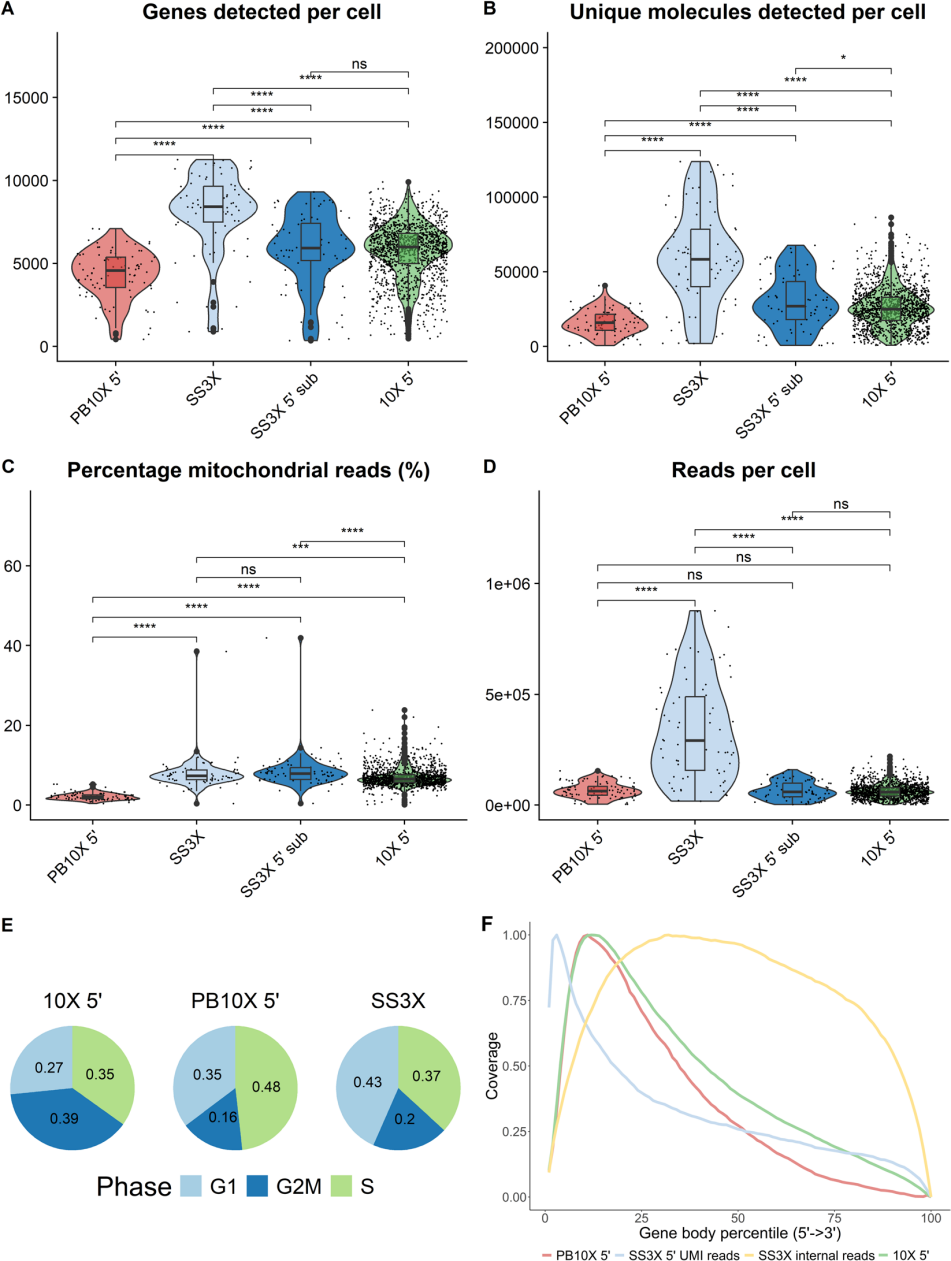
Comparative benchmarking results. **A** the number of detected genes per cell, (**B**) the number of unique molecules detected per cell, (**C**) the percentage of reads mapping to mitochondrial genes per cell, (**D**) the number of read pairs per cell, (**E**) the distribution of cells in the G1, G2/M, and S cell cycle phases across the different strategies, and (**F**) the gene body phases across the different strategies. Legend: Red: PB10X 5’, PB10X coupled to 10X Single Cell 5’ Gene Expression library construction; light blue: SS3X 5’ UMI reads; yellow: SS3X internal reads; green: 10X 5’, droplet-based 10X coupled to 10X Single Cell 5’ Gene Expression library construction. The result of the Wilcoxon t-test performed for each comparison is reported at the top. ns: *P* > 0.05; * *P* ≤ 0.05, ** *P* ≤ 0.01, *** *P* ≤ 0.001, **** *P* ≤ 0.0001

As illustrated in the upper right panel of Fig. 2, using the PB10X coupled to 10X Single Cell 5’ Gene Expression library sequencing dataset, without V(D)J enrichment, TRUST4 successfully retrieved the complete and productive TCR β-chain for 15.3% of the cells. TRUST4 could not reconstruct the TCR α-chain in this case, potentially indicative for lower TCR α-chain expression. As a remark, the TCR recovery rates for all discussed strategies are based on clonal Jurkat T lymphoblasts. In biological settings characterized by high TCR diversity, sensitive enrichment methods such as the 5 PB10X'-V(D)J- strategy are favored in single-cell settings to ensure accurate capture of TCR sequences from each cell without the need for deep sequencing of every cell.

### Benchmarking the performance of PB10X 5’ to SS3X and 10X 5’

The performance of PB10X coupled to 10X Single Cell 5’ Gene Expression library construction (hereafter referred to as PB10X 5’) was compared to the two strategies upon which the protocol is based, i.e. (1) SS3X and (2) droplet-based 10X Chromium coupled to 10X Single Cell 5’ Gene Expression library (hereafter referred to as 10X 5’), using Jurkat T lymphoblasts [26]. The latter dataset was retrieved from Yamawaki et al. [26]. All three strategies are strand-specific, except the internal read fraction from SS3X. Importantly, they use UMIs to allow accurate quantification and address PCR-bias, an important feature which often limited earlier comparison between Smart-seq2 (SS2) (which lacks UMIs) and other scRNA-seq strategies [27–29]. After quality filtering, we retained 85 and 76 cells out of 96 sorted cells for PB10X 5’ and SS3X, respectively. For some comparisons between the datasets, we first extracted the 5’ UMI-reads from the SS3X dataset, followed by subsampling to equal read depth across the different methods (hereafter referred to as SS3X 5’ sub), as illustrated in Fig. 3.

In terms of sensitivity, PB10X 5’ retrieved a mean of 4,343 genes and 16,137 UMIs per cell as illustrated in Fig. 3A, B. Consistent with previous research, SS3X retrieved the most genes and UMIs per cell in our benchmarking experiment (Supplementary Materials Table S2) [13]. Since the cDNA of PB10X 5’ was pooled from only 24 cells, the resulting yield of cDNA per pool was limited (4 ng – 41 ng). PB10X 5’ required more PCR pre-amplification cycles to obtain the required amount of cDNA for downstream library preparation than commonly used by 10X, possibly explaining the lower gene and UMI count per cell due to loss of complexity [30]. Nevertheless, the PB10X method is easily scalable, allowing for additional custom barcoded 10X-compatible TSOs, enabling pooling of more cells into a single cDNA pool. To note, for SS3X 5’ sub, no statistically significant difference compared to 10X 5’ in terms of the number of genes detected per cell was identified, while the difference in the number of detected UMIs remained statistically significant in favor of SS3X 5’ sub.

PB10X 5’ revealed the lowest mitochondrial fractions among the three methods. Earlier comparison between SS2 and 10X revealed 2.9–9.1 fold higher mitochondrial fractions for SS2, attributed to the more thorough disruption of organelle membranes by SS2 [27]. In our results shown in Fig. 3C, a similar trend was observed with higher proportions of mitochondrial genes for SS3X. However, the difference with 10X 5’ was not as pronounced as previously reported, which probably reflects the decrease in Triton X-100 concentration from 0.2% in SS2 to 0.1% in SS3X [13, 20]. One possible explanation for the lower mitochondrial fractions observed in PB10X is the inclusion of an AMPure bead-based purification step after pooling, which is not present in the SS3X workflow. This intermediate cleanup likely results in the preferential loss of shorter mitochondrial transcripts, many of which are under 200 nt and primarily of tRNA origin, thereby reducing their representation in the final PB10X libraries (Supplementary Fig. S4).

For the same batch of sorted Jurkat T lymphoblasts, PB10X 5’ showed higher proportions of cells in the S phase of the cell cycle and fewer cells in the G1 phase, as shown in Fig. 3E. However, as we only compare limited cell numbers, this might be coincidentally. Earlier comparisons did not find significant differences between SS2 and 10X 5’, however the former does not rely on UMI-counts [27]. Comparing the cell cycle scoring with the 10X 5’ dataset from Yamawaki et al. is difficult, as their Jurkat T lymphoblasts might be harvested in different conditions.

PB10X 5’ has the same limitations as 10X 5’ in terms of gene coverage and sensitivity. As the chemistry of 10X 5’ and PB10X is largely the same, both methods show a strong bias to the 5’ end of the gene body, while this bias is less pronounced for SS3X due to its use of both internal and 5’ UMI reads, as illustrated in Fig. 3F.

### PB10X 5’ has the most uniquely mapped reads and the highest proportion of bases mapping to coding regions

**Fig. 4:**
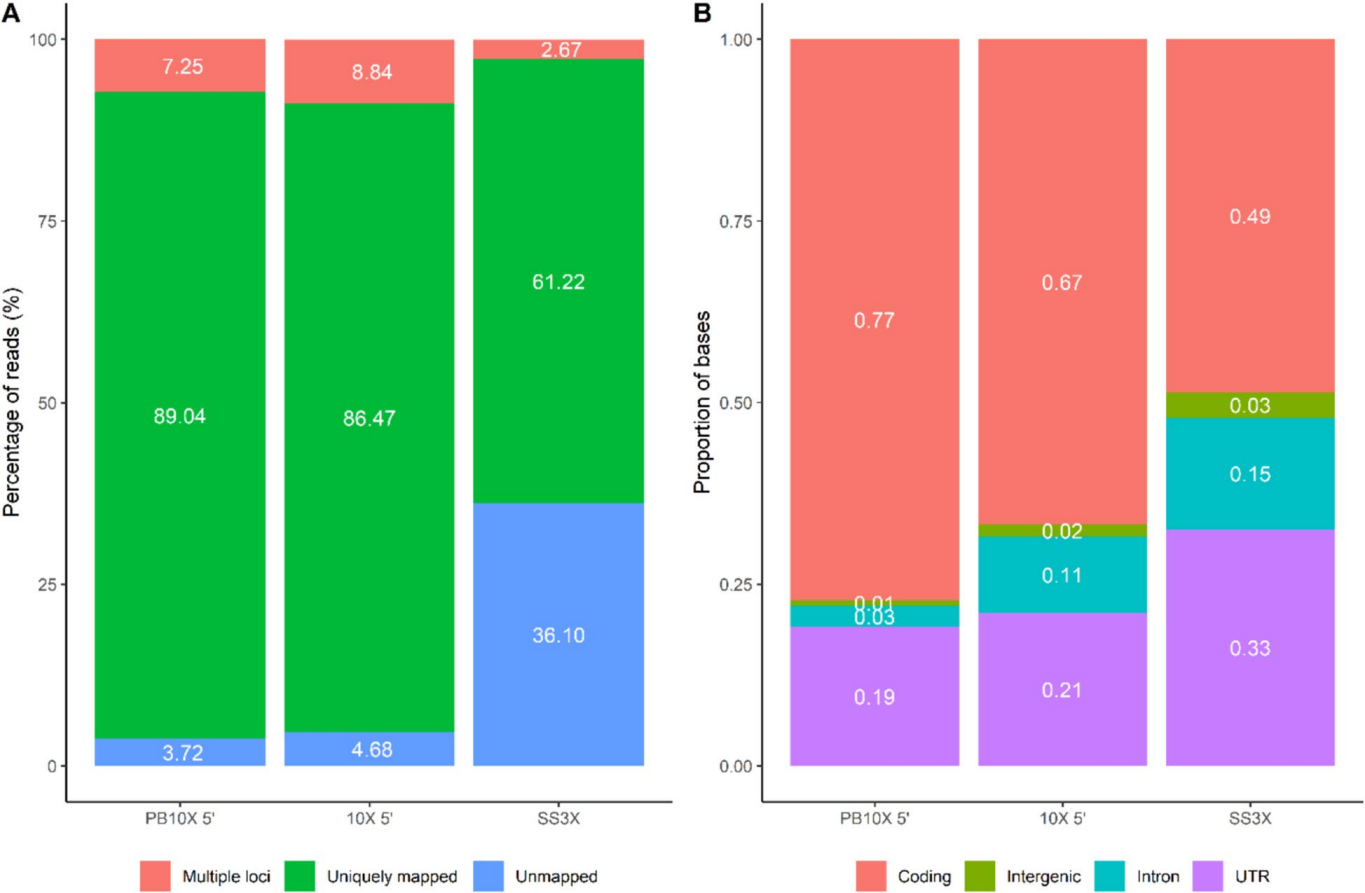
Overview of mapping statistics for the different strategies. **A** Percentage of reads assigned as multi-mapped, uniquely mapped and unmapped (including reads deemed too short and others) for the different strategies, as quantified using STAR implemented in zUMIs. **B** Percentage of reads that aligned to coding regions, intergenic regions, intronic regions and untranslated regions (UTR). PB10X 5’, PB10X coupled to 10X Single Cell 5’ Gene Expression library construction; SS3X, SS3X data including both 5’ UMI-reads and internal reads at full-depth (~ 338,000 reads/cell); 10X 5’, droplet-based 10X coupled to 10X Single Cell 5’ Gene Expression library construction

PB10X 5’ outperforms other methods in terms of mapping efficiency. Specifically, the PB10X 5’ strategy demonstrates the highest percentage of uniquely mapped reads and, consequently, the lowest percentage of unmapped reads compared to SS3X and 10X 5’, as illustrated in Fig. 4A. The elevated proportion of unmapped reads within SS3X data primarily results from reads that are too short to map (34.03%), with only a smaller fraction attributed to other unmapped read origins (2.07%). This distribution of mapping features aligns with the findings of Hagemann-Jensen et al. and might be attributed to the internal reads generated during the tagmentation process [13] Additionally, PB10X 5’ exhibits the highest proportions of bases mapping to coding regions, compared to SS3X and 10X 5’, as illustrated in Fig. 4B.

### PB10X 5’ and 10X 5’ reach sequencing saturation before SS3X

**Fig. 5:**
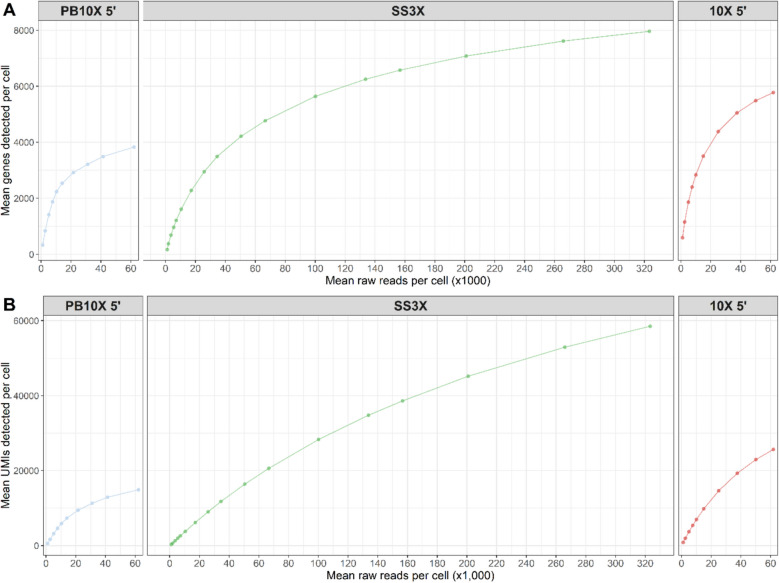
Sequencing saturation plots for PB10X 5’, SS3X, and 10X 5'. The raw reads for each strategy were subsampled to various levels targeting equal read depth per cell. (Panel **A**) Mean number of raw reads per cell plotted against the mean number of detected genes per cell. (Panel **B**) Mean number of raw reads per cell plotted against the mean number of detected unique molecular identifiers (UMIs) per cell. PB10X 5’, PB10X coupled to 10X Single Cell 5’ Gene Expression library construction; SS3X, SS3X data including both 5’ UMI-reads and internal reads at full-depth (~ 338,000 reads/cell); 10X 5’, droplet-based 10X coupled to 10X Single Cell 5’ Gene Expression library construction

To analyze library complexity and to determine the required sequencing depth, all three strategies were subjected to systematic subsampling. The number of unique genes and UMI-containing transcripts detected at various sequencing depths is illustrated in Fig. 5. Additionally, we calculated sequencing saturation as 1 minus the ratio of the number of unique counts divided by the total read count, resulting in a mean of 0.75, 0.80, and 0.58 for PB10X 5’, SS3X, and 10X 5’, respectively. Achieving sufficient sequencing depth for scRNA-seq is an empirical process, influenced by cell type and library preparation method. However, it is a crucial feature, as it was shown before that the proportion of genes exhibiting overdispersion, i.e. variance in counts exceeding Poisson distribution in cell-line scRNA-seq datasets, is related to the sequencing depth [31]. PB10X 5’ reached its plateau-phase at the lowest sequencing depth, followed by 10X 5’, and SS3X. Due to the presence of non-UMI-containing internal reads in the SS3X data, the detection of genes and UMIs initially lags other methods at lower sequencing depths. However, at higher sequencing depths per cell, SS3X excels and surpasses the other methods. It is important to note that scRNA-seq strategies with efficient cDNA pre-amplification require fewer reads to result in the same numbers of unique UMIs [32]. As mentioned above, the number of cDNA pre-amplification cycles in PB10X 5’ depends on the number of unique barcoded TSOs available per library pool. Increasing the number of unique TSOs per pool, and thus reduced cDNA pre-amplification cycles, will most likely increase library complexity. Regarding the optimal trade-off between sequencing depth and how many cells should be profiled, Zhang et al. mathematically demonstrated that for 3’ end scRNA-seq strategies, accounting for a fixed sequencing budget, sequencing as many cells as possible while reassuring that at least 1 UMI count for genes of biological interest is detected, is the most favorable trade-off [33]. For 5’-end strategies, such as PB10X 5’ and 10X 5’, the same can be assumed. However, PB10X solves the prior trade-off by allowing to allocate the available sequencing reads to those cells priorly identified as being of interest during indexed FACS and deep sequencing of these cells, while retaining downstream compatibility with any 10X Single Cell 5’ library construction kit.

### PB10X 5’ has similar dropout ratios as SS3X 5’ sub

**Fig. 6:**
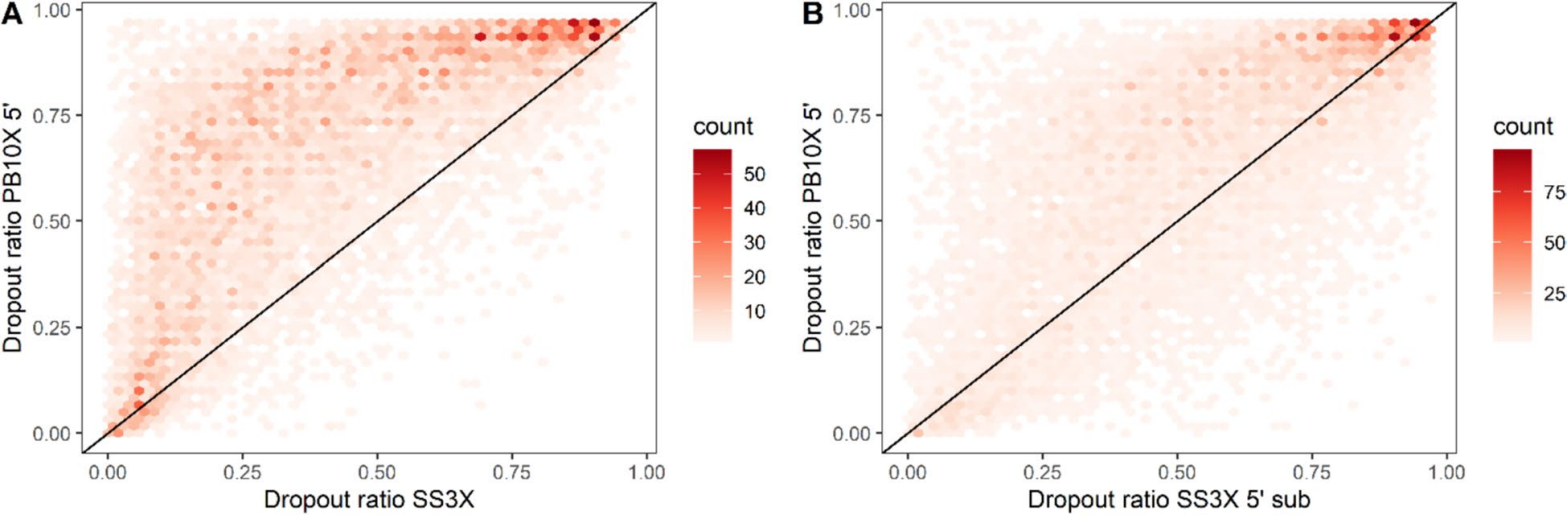
Comparison of the dropout ratios observed in PB10X 5’, SS3X, and SS3X 5’ sub. Each dot represents a gene. The dropout ratio is defined as the number of cells with zero counts for a specific gene divided by the number of total cells. A dropout ratio of 1 denotes that for none of the cells the gene has been detected. The diagonal line indicates equal dropout ratios between the compared strategies. (Panel **A**) Comparison of dropout ratios between PB10X 5’ and SS3X. (Panel **B**) Comparison of dropout ratios between PB10X 5’ and SS3X 5’ sub. PB10X 5’, PB10X coupled to 10X Single Cell 5’ Gene Expression library construction; SS3X, SS3X data including both 5’ UMI-reads and internal reads at full-depth (~ 338,000 reads/cell); SS3X 5’ sub, SS3X data including only 5’ UMI-reads and subsampled to equal read depth as PB10X 5’

Another important consideration in scRNA-seq is dropout, characterized by the observation that a gene is detected in one cell but not in another of the same cell type. These zero gene counts, whether arising from biological sources (e.g., gene regulation) or technical issues (e.g., mRNA capture efficiency during reverse transcription), lead to many genes being undetected, significantly contributing to the sparsity of scRNA-seq data [23, 34, 35]. While we studied a clonal Jurkat T lymphoblast cell line with the same phenotype, gene expression of a particular cell can still be influenced by many factors, e.g. cell cycle. To evaluate the dropouts in PB10X 5’, we compared its dropout ratio, defined as the proportion of cells with zero counts for a specific gene divided by the number of total cells, with SS3X and SS3X 5’ sub. Notably, these cell types originated from the same batch of sorted cells. As illustrated in Fig. 6A, based on the mutually present genes between the strategies, PB10X 5’ has higher dropout ratios than SS3X. However, this discrepancy improves if we only account for 5’ UMI-reads of SS3X and subsample to equal read depth, as illustrated in Fig. 6B. Similar conclusions were obtained by *Wang *et al*.* for the comparison between SS2 and 10X [27].

### PB10X 5’ resolves the strand invasion problem observed for SS3X

Strand invasion is a critical challenge in first-strand cDNA synthesis. This issue occurs when the TSO hybridizes with complementary regions within the first-strand cDNA before reaching the end of the mRNA, prematurely completing reverse transcription [13, 34, 36]. Consequently, strand invasion results in shortened cDNA, leading to biased UMI-counts and isoform detection [34, 35].

**Fig. 7:**
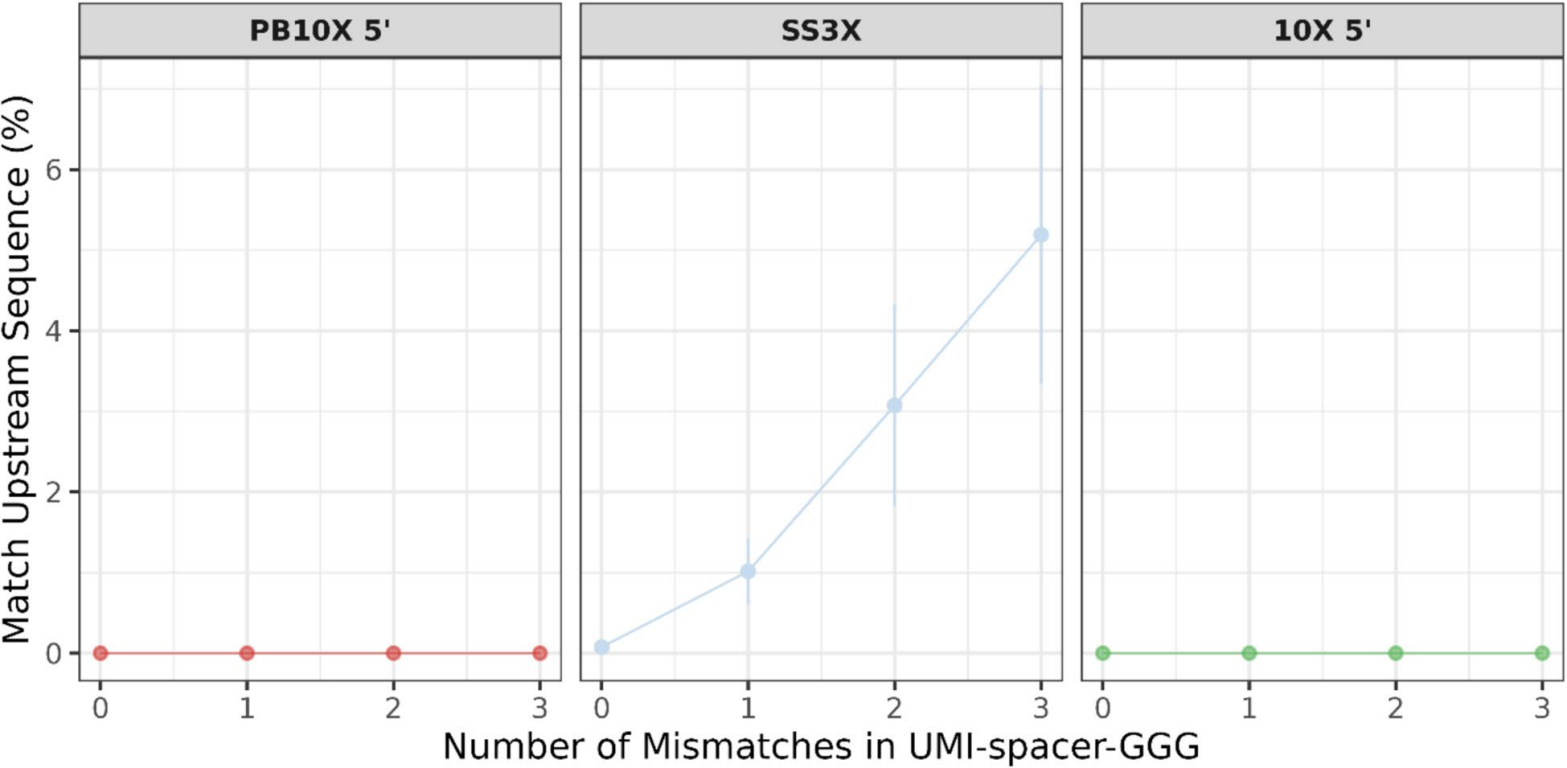
TSO strand invasion artifacts across the different methods. Percentage of deduplicated 5’ UMI-reads harboring a match between the UMI-SPACER-rGrGrG pattern and the 23-bp window of the reference genome sequence upstream of the alignment start position, with 0–3 mismatches. PB10X 5’, PB10X coupled to 10X Single Cell 5’ Gene Expression library construction; SS3X, SS3X data including both 5’ UMI-reads and internal reads at full-depth (~ 338,000 reads/cell); 10X 5’, droplet-based 10X coupled to 10X Single Cell 5’ Gene Expression library construction

In SS3X, the TSO was optimized to minimize strand invasion artifacts by introducing a WW-spacer sequence, in which W can be an A or T nucleotide. Although their improved TSO design represented a substantial enhancement over the original Smart-seq3 TSO, strand-invasion artifacts remain prominent in SS3X, as confirmed in this study [13] and illustrated in Fig. 7. In addition, increased percentages of intergenic reads, which may indicate strand invasion [35], were observed in SS3X compared to the other two strategies, as shown in Fig. 4. The strand-invasion artifacts are identified by looking at the percentage of deduplicated 5’ UMI-reads harboring a match between the UMI-SPACER-rGrGrG pattern and the 23-bp window of the reference genome sequence upstream of the alignment start position, with 0–3 mismatches.

In contrast, PB10X 5’ and 10X 5’ effectively eliminate these problematic artifacts. Two main reasons explain this difference. Firstly, the (PB)10X spacer (TTTCTTATAT) is likely more effective in preventing strand invasion compared to the SS3X spacer [13]. Secondly, the bead purification step after pooling first-strand cDNA libraries likely plays a crucial role in eliminating strand invasion, since an excess of TSOs can contribute to strand invasion. However, incorporating this purification step for SS3X is not possible as pooling cannot be performed yet at this step.

## Discussion

PB10X is a novel plate-based 10X-compatible 5’-end scRNA-seq technique that allows for the parallel analysis of both gene expression and enriched V(D)J amplicons of indexed sorted cells. The use of custom barcoded 10X-compatible TSOs enables downstream processing with a single pool, significantly enhancing time-efficiency, and scalability compared to methods like SS3X, which processes one library per cell [13]. In terms of cost-effectiveness, our quantitative analysis (Supplementary Materials File S2 and Supplementary Materials Fig. S5) demonstrates that PB10X is cost-effective compared to SS3X and the standard 5′ 10X Genomics workflow, if the number of barcoded TSOs is optimized for the number of cells processed. The scenarios simulated in Supplementary Materials File S2 include varying cell input sizes (96 vs. 384 cells), library types (V(D)J-only, gene expression-only, or combined), and TSO input scales (24, 96, or 384 uniquely barcoded TSOs). While PB10X consistently emerged as the most cost-effective solution in the 96 and 384 uniquely barcoded TSO scale, SS3X remains the cheapest option at 24 uniquely barcoded TSOs scale. In contrast, the 5′ 10X Genomics workflow remains by far the most expensive option at these low-throughput scales. As input size increases, however, the per-cell cost of 10X Genomics decreases, making it more attractive for high-throughput applications. A key factor influencing PB10X cost is the number of barcoded TSOs used. Increasing the number of TSOs reduces the number of downstream 10X library reactions required, thereby shifting the cost structure. These custom biotin-blocked, RNase-free, HPLC-purified TSOs cost approximately €103.5 each, but can be reused across multiple experiments, making them a worthwhile long-term investment in miniaturized workflows. The decision to order more TSOs can be tailored to the experimental design and anticipated throughput. Sequencing costs are also favourable for PB10X, with recommended read depths of just 20,000 reads per cell for gene expression and 5,000 reads per cell for V(D)J sequencing, further contributing to its overall cost-efficiency. PB10X-generated cDNA libraries are in principle compatible with any downstream commercially available 10X Single Cell 5’ library construction kit, however the use of the 10X Single Cell Human/Mouse BCR Amplification kit for BCR sequence repertoire purposes and the 10X Single Cell 5’ Feature Barcode Technology have not been experimentally tested yet. Beyond that, the PB10X method is also compatible with the Cell Ranger pipeline. A concrete example that highlights the practical value of PB10X is e.g. its potential application in the context of tumor-infiltrating lymphocyte (TIL) therapy. TIL-based adoptive T-cell therapy relies on isolating and expanding tumor-reactive T-cells from resected tumor material. In recent antigen-agnostic strategies, scRNA-seq is used to directly profile gene expression and TCR clonotypes of enriched TILs to identify dominant, tumor-reactive clonotypes [37]. PB10X would be particularly well suited to such workflows, as it allows for the efficient recovery of paired TCRαβ sequences from limited numbers of sorted CD3⁺CD45⁺ cells, common in small or low-yield biopsies, while remaining compatible with standard and robust 10X library kits. Its cost-efficiency at low cell input and low sequencing depth makes it an attractive option for studies constrained by limited tumor material or budget. Moreover, by using indexed FACS, direct pairing the individual cell’s transcriptome with additional TIL phenotype markers, e.g. FoxP3 and/or BTLA, is possible, and cannot be achieved using 10X without additional sample prep modifications.

### Advantages and limitations

The choice for a particular scRNA-seq strategy from the plethora of available scRNA-seq strategies currently available, both commercial and academic, is largely dependent on the application and the budget available (Supplementary Materials Table S3). To the best of our knowledge, PB10X is the first plate-based 10X-compatible strategy, offering a miniaturized workflow and the opportunity to start from limited numbers of indexed sorted single cells. While several components of PB10X individually draw from existing methodologies, the key innovation of PB10X lies in the integration of these features into a cohesive, scalable workflow that uniquely combines: (i) a barcoded, plate-based 5′ capture system compatible with standard, robust 10X library preparation kits, (ii) a post-barcoding pooling step that eliminates the need for processing each single-cell library individually through all downstream steps and enables streamlined purification prior to amplification. Indeed while other research (e.g. TIRTL-seq) and commercial protocols (e.g. SMARTer® Human scTCR α/β Profiling Kit) have shown that TCR-sequencing from FACS-isolated single cells in a plate-based workflow is possible, they do not offer the practical advantage of early pooling [38], (iii) mitigation of strand invasion and concatemerization via both a 5′-blocked TSO and an intermediate cleanup step, and (iv) direct pairing of upstream FACS-indexed sorting data with scRNA-seq data and compatibility with limited cell populations. This combination enables high-throughput capture of rare, phenotypically defined single cells with significantly reduced library complexity artifacts and hands-on time, benefits not achieved by SS3X or 10X alone. We showcased its excellent performance for TCR-sequencing and its applicability for gene expression profiling in low-throughput settings. While SS3X demonstrated comparable TCR-sequence recovery through *in silico* reconstruction, it necessitated an average of 338,000 reads per cell to achieve a paired TCRαβ success rate of 86.84%. This corresponds to an approximately 60-fold higher read depth per cell compared to what is required for PB10X 5’V(D)J-enriched libraries to achieve similar results. Nevertheless, the former generates the TCR-sequence data along the gene expression data, while PB10X necessitates two different library construction kits and independent sequencing. Furthermore, PB10X has the highest number of uniquely mapping reads and solves the issue of strand-invasion present in Smart-seq related protocols thanks to its TSO design and the intermediate purification.

As for limitations of PB10X, the number of detected genes and unique molecules per cell were lower than for the other strategies, probably related to limited number of unique TSOs used, resulting in the need for more PCR pre-amplification cycles. Additionally, differences in the oligod(T) primer sequence composition and the oligod(T)/TSO reaction concentrations between PB10X 5’ and regular 10X 5’ may have contributed to the differences in gene expression performance observed. The primer conditions in droplet-based 10X 5’ are presumably more optimized for downstream 10X profiling. Finally, we cannot exclude that during the purification upon completion of the RT-step, a fraction of the RT-products may not have been retained during pooling and purification. For larger scale projects, droplet-based 10X or other high-throughput technologies remain the preferred strategies compared to PB10X and SS3X. Additionally, we also note that the loss of cells during FACS-mediated single-cell isolation should be accounted for during experimental design, both for PB10X and SS3X. Even for regular 10X, FACS is often used during pre-processing to obtain a specific population of cells prior to loading the 10X microfluidics controller. Both the number of cells in the single cell suspension to be sorted, the event rate during sorting, and the percentage of cells in the target gate impact the extent of cell loss. However, we note that for 10X additional cell loss is expected due to cells not being incorporated into the GEMs. Along with the PB10X strategy, we also provided benchmarking between PB10X, 10X, and SS3X. As far as we are aware, no independent benchmarking between SS3X and 10X 5’ chemistry has been published yet.

## Conclusion

In conclusion, we present PB10X as a novel, plate-based, 10X-compatible 5’-end scRNA-seq strategy that can utilize indexed FACS to analyze ultra-rare cell populations. Unlike SS3X, PB10X pools the libraries early in the protocol, which reduces hands-on time and enables the inclusion of a purification step to effectively solve strand invasion. We profiled indexed sorted Jurkat T lymphoblasts and showcased PB10X’s efficacy in generating gene expression and V(D)J-enriched sequencing libraries. Complementary, we provide comprehensive benchmarking comparisons between PB10X, 10X, and SS3X. Albeit being less sensitive than SS3X, PB10X combined with the gene expression library construction kit detects a mean of 4,343 and 16,137 genes and UMIs per cell, respectively. PB10X combined with V(D)J library construction enabled efficient retrieval of paired alpha and beta TCR chains in 81.71% of cells at lower sequencing depth than SS3X. This novel approach holds promise for advancing single-cell transcriptomics research in settings where only small populations of sorted cells are available.

## Methods

### Cell sources, culturing, and sorting

Jurkat T lymphoblasts (clone E6-1) were purchased from the DSMZ repository (Braunschweig Germany) and cultured in RPMI 1640 medium (Gibco) supplemented with 10% fetal bovine serum (FBS, Hyclone) and 2 mM L-glutamine (Gibco) at 37 °C and 5% CO2. Jurkat T lymphoblasts were first blocked with anti-human FcR (Miltenyi, #130–059–901) for 10 min at room temperature to avoid non-specific binding of antibodies. Next, cells were incubated with antibodies against CD3 (clone UCHT1, BioLegend cat. Nr. 300,440, FITC, dilution: 1:200) and TCR α/β (clone IP26, BioLegend cat. Nr. 306,718, APC, dilution: 1:200). After a wash in phosphate buffered saline (PBS, Gibco), cells were resuspended in PBS. Propidium iodide was used to mark dead cells. CD3 + TCRαβ + single, live cells were sorted as single cells into 384-well plates containing the protocol-specific lysis mix, using BD FACSAria Fusion (BD Biosciences) with a 70-µm nozzle. Sorting purity was 98%. After sorting, each plate was immediately sealed, centrifuged, and stored at −80 °C upon further processing.

### Liquid handling

For handling nanoliter-scale volumes, the I.DOT (Dispendix) was used as a non-contact liquid dispenser.

### RNase-free handling

Throughout the experiments, nuclease-free water was exclusively utilized. Only plates and tubes certified as nuclease-free were used. Additionally, all subsequent procedures were performed on work surfaces and with equipment cleaned with RNaseZAP™.

### SS3X on 96 Jurkat T lymphoblasts

Library preparation was carried out with the improved version V.2. of the SS3X protocol as deposited on protocols.io. Only minor adjustments were made in comparison with this version of the protocol. 12 cycles of pre-amplification PCR were performed. Modifications include adjusting the input of Tn5 transposase enzyme (Diagenode) to 0.014 µL per reaction. Additionally, the use of 0.2% SDS to stop the tagmentation reaction and the inclusion of 0.025% Tween-20 within the index PCR mix to counteract the effects of SDS were omitted. Furthermore, the index PCR mix was refined by utilizing only 0.1 µM/each of each index primer, and 0.750 ng of tRNA carrier (ThermoFisher) was added to each reaction to enhance yield, before performing 15 PCR-cycles.

### PB10X cDNA generation of 96 Jurkat T lymphoblasts

Cells were sorted similar to the SS3X protocol into a prefilled 384-well plate with each well containing 300 nL of the PB10X lysis mix and covered with a 3 µL silicon oil (Sigma-Aldrich) overlay. The PB10X lysis mix comprises 5% PEG 8000 (Sigma-Aldrich), 0.10% Triton X-100 (Sigma-Aldrich), 0.5 U/µL Recombinant RNase Inhibitor (Takara), 0.125 µM oligodT30VN primer (5’-Biotin-ACGAGCATCAGCAGCATACGATTTTTTTTTTTTTTTTTTTTTTTTTTTTTTVN-3’, IDT), 0.5 mM dNTPs/each (ThermoFisher) and 0.75 µM of custom barcoded 10X-compatible TSOs (IDT, Supplementary Materials Table S1). Volumes of PEG 8000, oligodT30VN primer, dNTPs each and the TSOs were adjusted to the reverse transcription (RT) volume, as performed in the SS3X protocol. Following dispensing, lysis plates underwent brief centrifugation to ensure proper storage of the lysis mix under the oil overlay at −80°C.

Stored plates of sorted cells were denatured at 72 °C for 10 min. Immediately following denaturation, 0.1 µL of the RT mix, composed of 25 mM TrisHCl pH 8.4 (Alfa Aesar), 30 mM NaCl (ThermoFisher), 2.5 mM MgCl_2_ (ThermoFisher), 8 mM DTT (ThermoFisher), 1.0 mM GTP (ThermoFisher), 0.25 U µL^−1^ Recombinant RNase inhibitor (Takara), and 2 U µL^−1^ Maxima H Minus reverse transcriptase (ThermoFisher), was dispensed to the wells. The plate was briefly centrifuged after dispensing the RT mix. RT was performed at 42 °C for 90 min, followed by ten cycles of 50 °C for 2 min and 42 °C for 2 min, followed by 5 min at 85 °C. Hereafter, pooling was performed for every 24 different TSOs. The oil phase was discarded, followed by 0.6X AMPureXP bead (Beckman Coulter) purification with final elution in nuclease-free water to obtain a final volume of 9.6 µL per pool.

Finally, a PCR pre-amplification was conducted, adding 14.4 µL PCR mix per pool. The PCR mix consists of 1X SeqAmp buffer (Takara), 0.025 U µL^−1^ SeqAmp Polymerase (Takara), 0.5 µM 10X forward primer (5’-CTACACGACGCTCTTCCGATCT-3’, IDT) and 0.1 µM SS3X reverse primer (5’-ACGAGCATCAGCAGCATAC*G*A-3’, IDT). PCR pre-amplification was performed as follows: initial denaturation at 95 °C for 1 min, followed by 18 cycles of 98 °C for 10 s, 65 °C for 30 s and 68 °C for 4 min. The final elongation step was conducted at 72 °C for 10 min.

After PCR pre-amplification, all 4 pools were purified using 0.7X AMPureXP beads ratio and eluted in 20 µL of nuclease-free water. Consequently, the quality and quantity of the cDNA libraries were checked with the Fragment Analyzer (Agilent) using the High Sensitivity NGS Fragment Analysis kit (1 bp – 6000 bp, Agilent).

### 10X Single Cell 5’ Gene Expression library construction

The PB10X-compatible cDNA libraries were further processed with the 10X Library Construction kit PN1000190 (10X Genomics) and the Dual Index TT Set A PN-1000215 (10X Genomics), following the 10X 5’ Gene Expression Dual Index Library construction protocol. The required volumes were halved while maintaining all prescribed concentrations as specified in the protocol. An input of 4–5 ng cDNA per pool was used. The final sample index PCR was carried out using 16 cycles of amplification.

### 10X Single Cell 5’ V(D)J library construction

The PB10X-compatible cDNA libraries were further processed with the Chromium Single-cell Human TCR Amplification kit PN-1000252 (10X Genomics), the 10X Library Construction kit PN1000190 (10X Genomics), and the Dual Index TT Set A PN-1000215 (10X Genomics), following the 10X 5’ V(D)J Amplification and V(D)J Dual Index Library construction protocol. The required volumes were halved while maintaining all prescribed concentrations as specified in the protocol. 0.5 ng of cDNA per pool was used as input for the first V(D)J amplification step. The Human T-Cell Mix 1 and 2 v2 (10X Genomics) were utilized specifically for V(D)J amplifications. The first V(D)J amplification was carried out using 12 cycles, while the second V(D)J amplification was carried out using 10 cycles. For the V(D)J library construction, a minimum input of 6 ng V(D)J amplified cDNA was employed, and a total of 8 cycles was applied for the sample index PCR.

### Sequencing

The PB10X libraries were sequenced across two AVITI™ sequencer runs, reading 26 nt for read 1, 90 nt for read 2, 10 nt for P7 index and 10 nt for P5 index. An average target of 50,000 reads per cell was aimed for gene expression and 5,000 reads per cell for immune profiling. The SS3X libraries were also sequenced on two AVITI™ sequencer runs, generating PE150 reads, with a target of 300,000 reads per cell.

### Analysis of SS3X Jurkat T lymphoblast data

The raw.fastq files were processed using zUMIs v.2.9.7e [39]. The GRCh38 release 111 was used as a reference.

### Analysis of PB10X Jurkat T lymphoblast data

The per pool demultiplexed raw.fastq files were first analyzed using Cell Ranger v7.2.0 using default settings and the reference transcriptome based on GRCh38 Ensembl 111 release, generated using the Cell Ranger mkref command [5]. For TCR-profiling, we ran the Cell Ranger V(D)J pipeline for each of the pools using the default settings. Subsequently, the Cell Ranger output of the 4 pools was aggregated using Cell Ranger aggr with –normalize = none. The resulting filtered_feature_bc_matrix files were used for downstream analysis. The number of reads per barcode was extracted from the possorted_genome_bam.bam using samtools view possorted_genome_bam.bam | grep CB:Z: | sed 's/.*CB:Z:[ACGT]*.*/\1/' | sort | uniq -c > reads_per_barcode.txt, with samtools v1.6 [40]. It should be noted that due to IDT’s standard 60-nt synthesis, the PB10X TSO uses a UMI shortened by one nucleotide compared to the standard 10X design. Cell Ranger reads the first spacer base as part of the UMI, but since this base is constant, it doesn't affect compatibility. To avoid UMI shortening, custom synthesis beyond the standard 60-nt limit could be considered using alternative synthesis options.

### Re-analysis of the 10X 5’ Jurkat T lymphoblast data from Yamawaki et al

The sequencing data of *Yamawaki *et al*.* [26], annotated as 10X 5’ in the main text, was reanalyzed using Cell Ranger version v7.2.0. The original raw sequencing data from sample GSM4987144: 05_Chromium_5p_v1_lymphocyte_cell line_mix was downloaded from the sequencing read archive (SRA) using SRAtools with the command fasterq-dump –p –threads 20 –S, for runs SRR13296299, SRR13296300, SRR13296301, and SRR13296302. This data was generated using the Chromium Single Cell 5’ v1 kit (10X Genomics). Subsequently, Cell Ranger count was run on the downloaded.fastq files using the refdata-gex-GRCh38_and_GRCm39-2024-A transcriptome, as downloaded from the 10X Genomics website, using the default settings. The generated possorted_genome_bam.bam file was filtered for the Jurkat T lymphoblast barcodes using subset-bam [41], based on the list of barcodes of Jurkat T lymphoblasts as provided by *Yamawaki *et al*. (2021)*. The .fastq files from the Jurkat-filtered.bam file were extracted using Cell Ranger bamtofastq v1.4.1 [42]. Finally, the.fastq files were reprocessed with Cell Ranger 7.2.0 using default settings and the GRCh38 release 111 reference transcriptome, generated as described above. The resulting barcodes.tsv.gz, features.tsv.gz, and matrix.mtx.gz files were used as input for Seurat v5. The number of reads per barcode was extracted from the possorted_genome_bam.bam using samtools view possorted_genome_bam.bam | grep CB:Z: | sed 's/.*CB:Z:[ACGT]*.*/\1/' | sort | uniq -c > reads_per_barcode.txt, with samtools v1.6 [40].

### TSO concatemerization

To assess TSO concatemerization in standard short-read Illumina sequencing data, two times 96 wells of a 384 well-plate were filled with 10 pg of pre-extracted RNA from the LOUCY cell line (ACC 394, DSMZ). This plate was processed and pooled according to the PB10X protocol using a 5’-unblocked custom barcoded 10X-compatible TSO (5’-CTACACGACGCTCTTCCGATCTAAAGTAGTCAAGCCTANNNNNNNNNTTTCTTATATrGrGrG-3’, IDT) for the first 96 wells and a 5’-blocked custom barcoded 10X-compatible TSO (5’-Biotin-CTACACGACGCTCTTCCGATCTAAAGTAGTCAAGCCTANNNNNNNNNTTTCTTATATrGrGrG-3’, IDT) for the other 96 wells. Library construction of the obtained cDNA was performed using 10X Single Cell 5’ Gene Expression Dual Index Library construction protocol, followed by paired-end Illumina NovaSeq 6000 sequencing, with read 1 28 nucleotides and read 2 90 nucleotides. Uniquely mapped read percentages were calculated using STAR (version 2.7.10a) using the GRCh38 reference genome [43]. Additionally, TSO concatemers were specifically searched for and quantified using bbduk.sh literal = ATATGGGCTAC,GTAGCCCATAT k = 11 mm = f rcomp = f int = f, with bbduk v39.06 [44]. To reveal the sequences of these TSO concatemers specifically, a PB10X cDNA pool was generated from 96 wells filled with 10 pg of LOUCY RNA using the 5’-unblocked TSO. This cDNA pool was subsequently sequenced on a priorly used and washed PromethION R10.4.1 flow cell. The ONT library prep was performed as stated in the Ligation sequencing amplicons protocol in conjunction with the SQK-NBD114.24 kit (ONT). Briefly, 130 ng PB10X cDNA was used as input for the End-prep step, followed by Ampure XP-bead mediated clean-up in 1:1 ratio. Subsequently, 6 µL of the End-prepped cDNA (9.94 ng/µL) was used for native barcode ligation for 20 min at room temperature, followed by Ampure XP-bead mediated clean-up in a 0.4:1 ratio. Finally, the ONT Native adapter was ligated to the barcoded sample, and purified once more using the 0.4:1 Ampure XP beads to sample ratio. The final sequencing library was prepared by adding 100 µL of Sequencing Buffer and 68 µL of Library beads to the eluted library. The R10.4.1 PromethION flow cell was primed using Flow Cell Flush and 30 µL of Flow Cell Tether, before loading 200 µL of the final sequencing library onto the flow cell. Basecalling was performed using MinKNOW 23.07.12 in superhigh-accuracy mode.

### Subsampling of reads for comparison of PB10X and SS3X datasets

To permit an unbiased comparison, the SS3X dataset was subsampled to achieve an equal read depth per cell in some comparisons (denoted as SS3X 5’ sub in the main text). The 5’ UMI-reads were extracted prior to subsampling from the raw.fastq files using bbduk (v.38.84) [44] filtering in paired-end mode with literal = ATTGCGCAATG for read 1 with hdist = 1. Subsampling of the extracted reads was achieved by running seqtk sample -s 100. Synchronization of the.fastq files with their index.fastq files was performed using fastq_pair [45]. The synced subsampled.fastq files were used as input for zUMIs v.2.9.7e.

### Downstream analysis

Except for the specific analysis stated below, all downstream analyses were performed in R 4.3.3. The count data of all strategies were mainly analyzed using Seurat v5 [46–48]. The mitochondrial gene ids were extracted from EnsDb.Hsapiens.v86 using filter ‘MT’. Ensembl gene IDs and gene symbols were retrieved from the biomaRt package [49, 50]. We used the cell cycle genes as built into Seurat based on the work of Kowalczyk et al. [51]. An unpaired two-samples Wilcoxon t-test was performed to determine statistical differences between datasets. All other figures were made using the ggplot2 package [52].

### Mapping characteristics and gene body coverage

The raw.fastq files for each strategy were mapped using STAR as implemented in zUMIs using standard settings. The percentage of reads that was uniquely mapped, multimapped, and unmapped (too short & other) were extracted from the Log.final.out file. The proportion of bases mapped to coding, intronic, UTR, and intergenic loci were calculated using Picard CollectRnaSeqMetrics (v2.20.4) using the.bam file generated by zUMIs or Cell Ranger as input. RSeQC-5.0.3. was used to evaluate the gene body coverage for all strategies using the geneBody_coverage.py Python script.

### Construction of saturation curves

To check if all datasets achieved sequencing saturation, saturation curves were constructed. Sequencing saturation was studied by subsampling the raw .fastq files targeting an average of 1 K, 2.5K, 5 K, 7.5K, 10 K, 15 K, 25 K, 37.5 K, 50 K, and 75 K reads per cell for PB10X and 10X 5’, and an average of 1 K, 2.5K, 5 K, 7.5K, 10 K, 15 K, 25 K, 37.5 K, 50 K, 75 K, 100 K, 150 K, 200 K, 250 K, 300 K, 350 K, 400 K, 450 K, and 500 K reads per cell for SS3X. For PB10X, only the first pool of 21 cells was used as representative. The aforementioned thresholds were multiplied by the number of cells observed, and subsampling to that number of reads was achieved using seqtk -s 100. For SS3X, fastq_pair was used to synchronize the subsampled.fastq files of read1 and read2 with their respective.fastq index reads [45]. For PB10X and 10X 5’ of Yamawaki et al., Cell Ranger count was run for each subsampling level. For SS3X, equivalently, zUMIs was run for each of the subsampling levels. Additionally, sequencing saturation was calculated as 1 – (the number of unique counts/total read count).

### TCR reconstruction using TRUST4

TCR reconstruction based on the complete (both 5’ UMI and internal reads) SS3X was performed using TRUST4 (v1.1.2) [17], with flags –barcode BC –UMI UB –abnormalUnmapFlag. For the PB10X gene expression data, flags –barcode CB –UMI UB –abnormalUnmapFlag were used. The resulting barcode_airr.tsv and cdr3.out file were used as input in R4.3.3. using the ‘airr’ package (v1.5.0) [53]. During downstream analysis, only cells that had a complete, productive (no stop codon) TCR chain, and possessed a fully identified CDR3 region were considered for stringency.

### Strand invasion profiling

To profile the presence of strand invasion for all strategies, we used the R scripts and strategy published in the FLASH-seq paper, with slight adaptations [35]. In short, the UMI-length was set as 10, 9, and 10 nucleotides for SS3X, PB10X 5’, and 10X 5’, respectively. As SS3X encompasses a variable spacer for each transcript, annotated as WW, we extracted the UMIs in SS3X as having 10 nucleotides instead of 8 using zUMIs. The last two nucleotides were subsequently annotated as spacer. For PB10X 5’ and 10X 5’, the spacer is fixed (“TTTCTTATAT”), which permitted the use of the UMIs as extracted by Cell Ranger. As the latter spacer is significantly longer than the ones tested in the FLASH-seq paper, the first 23 (instead of 20) upstream nucleotides were considered for comparison with the UMI. Lastly, we used fuzzy matching using agrep to account for mismatches rather than taking only 5’ mismatches into account.

## Supplementary Information

Supplementary Material 1.

Supplementary Material 2.

## Data Availability

The data supporting the conclusions of this article is available in the NCBI Sequence Read Archive using accession number BioProject ID PRJNA1144900. The count tables for PB10X and SS3X can be found in the Gene Expression Omnibus (GEO) using accession number GSE294062.
