# Supplementary material for: Plate-based 10X Genomics-compatible single-cell RNA-sequencing based on Smart-seq3xpress
Source: BMC Genomics. 2025 Dec 6;27:32. doi: 10.1186/s12864-025-12286-2 (PMC12797766; doi:10.1186/s12864-025-12286-2)
Supplement: Supplementary file 1 — Supplementary Material 1. [file 12864_2025_12286_MOESM1_ESM.docx]

Supplementary materials

Table of Contents

**Supplementary Materials Table S1 Custom barcoded 10X-compatible TSO sequences………………………..2**

**Supplementary Materials Table S2 Per cell characteristics for PB10X, SS3X 5’, and 10X 5’……………………3**

**Supplementary Materials Table S3 Comparative Analysis of Methodological Features……………………..…4**

**Supplementary Materials Figure S1** **Fragment Analyzer electropherogram of pre-amplified library using a 5’-unblocked TSO…………………………………………………………………………………………………………………….5**

**Supplementary Materials Figure S2 Example of TSO concatemerization sequence………………………………6**

**Supplementary Materials Figure S3 Percentages of cells with successful TCR reconstruction across the different strategies after subsampling settings ………………………………..……………………………………………7-8**

**Supplementary Materials Figure S4 Percentage of reads mapping to short mitochondrial genes (< 200 nt) per cell across the different strategies………………………………………………………………………………………….9**

**Supplementary Materials Figure S5 Quantitative cost comparison of PB10X, SS3X and 5’ 10X Genomics workflows across different conditions……………………………………………………………………………………………….10**

**Supplementary Materials Table S1 Custom barcoded 10X-compatible TSO sequences.**

| TSO | Sequence 5’-3’ |
| --- | --- |
| 1 | 5’-Biotin-CTACACGACGCTCTTCCGATCTAGTGTCATCATGCATGNNNNNNNNNTTTCTTATATrGrGrG-3’ |
| 2 | 5’-Biotin-CTACACGACGCTCTTCCGATCTGCGCAGTAGTACGACGNNNNNNNNNTTTCTTATATrGrGrG-3’ |
| 3 | 5’-Biotin-CTACACGACGCTCTTCCGATCTGCGCAGTGTGCATCTANNNNNNNNNTTTCTTATATrGrGrG-3’ |
| 4 | 5’-Biotin-CTACACGACGCTCTTCCGATCTTCTCATACATGAGCGANNNNNNNNNTTTCTTATATrGrGrG-3’ |
| 5 | 5’-Biotin-CTACACGACGCTCTTCCGATCTTAGAGCTGTGATGATANNNNNNNNNTTTCTTATATrGrGrG-3’ |
| 6 | 5’-Biotin-CTACACGACGCTCTTCCGATCTCAGATCAGTATCAGTCNNNNNNNNNTTTCTTATATrGrGrG-3’ |
| 7 | 5’-Biotin-CTACACGACGCTCTTCCGATCTCAGAGAGAGTAGATGTNNNNNNNNNTTTCTTATATrGrGrG-3’ |
| 8 | 5’-Biotin-CTACACGACGCTCTTCCGATCTACTGCTCTCAGCACATNNNNNNNNNTTTCTTATATrGrGrG-3’ |
| 9 | 5’-Biotin-CTACACGACGCTCTTCCGATCTCTGTGCTCACACGCTGNNNNNNNNNTTTCTTATATrGrGrG-3’ |
| 10 | 5’-Biotin-CTACACGACGCTCTTCCGATCTGCTGCGAGTATATGAGNNNNNNNNNTTTCTTATATrGrGrG-3’ |
| 11 | 5’-Biotin-CTACACGACGCTCTTCCGATCTTGACTAGCATCAGTCANNNNNNNNNTTTCTTATATrGrGrG-3’ |
| 12 | 5’-Biotin-CTACACGACGCTCTTCCGATCTAGATCTGCACAGTCGCNNNNNNNNNTTTCTTATATrGrGrG-3’ |
| 13 | 5’-Biotin-CTACACGACGCTCTTCCGATCTCTCGTCACACGTCTCTNNNNNNNNNTTTCTTATATrGrGrG-3’ |
| 14 | 5’-Biotin-CTACACGACGCTCTTCCGATCTAGCTCTCTCACGCATANNNNNNNNNTTTCTTATATrGrGrG-3’ |
| 15 | 5’-Biotin-CTACACGACGCTCTTCCGATCTCTGATAGCATCGACGCNNNNNNNNNTTTCTTATATrGrGrG-3’ |
| 16 | 5’-Biotin-CTACACGACGCTCTTCCGATCTCTACGTCAGAGTACATNNNNNNNNNTTTCTTATATrGrGrG-3’ |
| 17 | 5’-Biotin-CTACACGACGCTCTTCCGATCTGCATGCGTCGAGAGCANNNNNNNNNTTTCTTATATrGrGrG-3’ |
| 18 | 5’-Biotin-CTACACGACGCTCTTCCGATCTTGCGCAGCATGACATCNNNNNNNNNTTTCTTATATrGrGrG-3’ |
| 19 | 5’-Biotin-CTACACGACGCTCTTCCGATCTGACAGAGTCAGCTCTCNNNNNNNNNTTTCTTATATrGrGrG-3’ |
| 20 | 5’-Biotin-CTACACGACGCTCTTCCGATCTCGCTATCAGCGACGTANNNNNNNNNTTTCTTATATrGrGrG-3’ |
| 21 | 5’-Biotin-CTACACGACGCTCTTCCGATCTGACGCGTAGCGATATANNNNNNNNNTTTCTTATATrGrGrG-3’ |
| 22 | 5’-Biotin-CTACACGACGCTCTTCCGATCTGATCGTACATCAGTACNNNNNNNNNTTTCTTATATrGrGrG-3’ |
| 23 | 5’-Biotin-CTACACGACGCTCTTCCGATCTACATACGTCGTCTGCTNNNNNNNNNTTTCTTATATrGrGrG-3’ |
| 24 | 5’-Biotin-CTACACGACGCTCTTCCGATCTAAAGTAGTCAAGCCTANNNNNNNNNTTTCTTATATrGrGrG-3’ |

**Supplementary Materials Table S2 Per cell characteristics for PB10X, SS3X 5’, and 10X 5’.**

All values shown are mean values per cell. For SS3X 5’ sub, first the 5’ UMI-reads of the SS3X dataset were extracted, before subsampling the number of these 5’ UMI-reads to about equal read depth as PB10X and 10X 5’. Cells with less than 200 detected genes and genes present in less than 3 cells were filtered out before summarizing these results.

|  | PB10X 5’ | SS3X | SS3X 5’ sub | 10X 5’ |
| --- | --- | --- | --- | --- |
| Mean genes per cell | 4,343 | 8,041 | 5,846 | 5,775 |
| Mean UMI counts per cell | 16,137 | 59,846 | 30,191 | 25,650 |
| Mean percentage mapped to mitochondrial genes (%) | 2.15 | 7.94 | 8.43 | 6.97 |
| Mean reads per cell | 66,700 | 338,173 | 65,645 | 61,778 |

**Supplementary Materials Table S3 Comparative Analysis of Methodological Features.**

Comparison of PB10X, SS3X, and 5’ 10X Genomics scRNA-seq techniques.

|  | PB10X | Smart-seq3xpress | 5’ 10X Genomics |
| --- | --- | --- | --- |
| Cell Isolation Strategy | FACS-based | FACS-based | Droplet-based |
| Phenotypic information | Available from FACS staining and indexed sorting | Available from FACS staining and indexed sorting | No phenotypic information of individual cells available |
| Cellular input | One cell/well in 384-well plate, multiple plates can be multiplexed | One cell/well in 384-well plate, multiple plates can be multiplexed | ≥ 1,000 cells per run  Multiple runs may be done in parallel  Optimal input cell concentration is 700-1,200 cells/µL |
| 5’ TSO blocking strategy | 5’ biotin-blocking group | 5’ biotin-blocking group | 5’ 10X bead |
| SPACER for strand invasion | TTTCTTATAT | WW | TTTCTTATAT |
| cDNA Barcoding | RT with template switching  Cell-specific barcode and UMI within TSO | RT with template switching  UMI within TSO  Cell-specific barcode incorporated within final sample index PCR step | RT with template switching  Cell-specific barcode and UMI within TSO on the bead |
| Pooling strategy | Post-RT | Post-index PCR | Post-RT |
| Recommended minimum sequencing depth | Min. 20 x 10^3^ reads/cell for gene expression  Min. 5 x 10^3^ reads/cell for immune profiling | Min. 1 x 10^5^ reads/cell | Min. 20 x 10^3^ reads/cell for gene expression  Min. 5 x 10^3^ reads/cell for immune profiling |
| Sequencing Type | Paired-end, Dual Indexing | Paired-end, Dual Indexing | Paired-end, Dual Indexing |
| Gene expression information | Possible, use of 10X Single Cell 5’ Gene Expression library construction kit, 5’-end reads only | Full-length transcript coverage: 5’-end reads + internal reads | Possible, use of 10X Single Cell 5’ Gene Expression library construction kit, 5’-end reads only |
| V(D)J information | Possible, use of 10X Single Cell 5’ V(D)J library construction kit, TCR/BCR-enriched cDNA library | No specific targeted TCR/BCR-enrichment  TCR reconstruction possible with internal reads using TRUST4 | Possible, use of 10X Single Cell 5’ V(D)J library construction kit, TCR/BCR-enriched cDNA library |
| Splicing information | Not possible with short-read sequencing | Partially possible with short-read sequencing | Not possible with short-read sequencing |


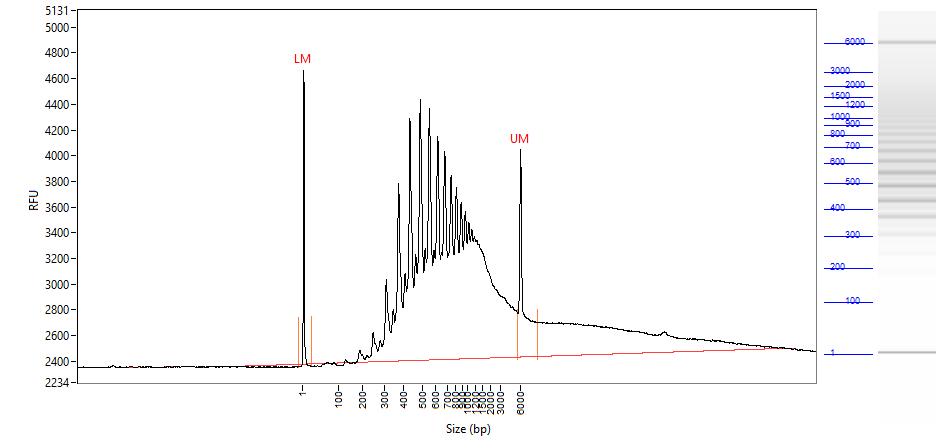
**Supplementary Materials Figure S1** **Fragment Analyzer electropherogram of pre-amplified library using a 5’-unblocked TSO.**

Data from 96 pooled wells each filled with 10 pg of pre-extracted RNA from the LOUCY cell line prepped using a 5’-unblocked TSO causing a ‘hedgehog’ pattern indicating TSO concatamerization.

@7a7cdaa0-0b06-4f12-893b-f812b0e5d59e runid=d8df71698204f9a649acb19518d09c563f01c489 sampleid=no_sample read=140182 ch=569 start_time=2023-12-09T08:15:57Z model_version_id=dna_r10.4.1_e8.2_400bps_sup@v4.2.0 barcode=barcode12

CTACACGACACTCTCCGATCTAAAGTAGTCAAGCCTAGGCTCAAAATTTCTTATATGGGCTACACGACGCTCTACACGACGCTCTTCCGATCTAAAGTAGTCAAGCCTAGAACACGAATTTCTTATATGGGCTACACGACGCTCTTCCGATAAAAGTGGTCGACACGTTCAAAATTTCTTATATGGGCTACACGACGCTCTACACGACGCTCTTCCGATCTAAAGTAGTCAAGCCTAGGAAGAGAATTTCTTATATGGGAGAGCGTCACATGTAGGCCCATATAAGAAATCCTTGTTCTAGGCTTGACTACTTTAGATCAAAAACATAGTGTAGCCCCGTATAGAAATTCGTGTTCTAGGCTTGACTACTTTACGATCGGAAGAGCGTCGTGTAGACGTCGTGTAGCCCATATAAGAAATTTTAGAGCCTAGGCTTGACTACTTAGATCGGAAGAGCGCTAGTGTAGCCCATATAGAGGTACCGTTGTTGTAGGCTTGACTACTTTTAGATCGGAAGAGCGTCGTGTAGCCCCATAGAAATTCGTGTTCTAGGCTTGACTACTTTAGATCGGAAGAGCGTCGTGTAGAGCGTCGTGTAGCCCATATAAGAAATTTTGAGCCTAGGCTTGACTACTTTAGATCGGAAGAGCGTCGTGTAGAGCGTCGTGTAGCCCATATAAGAAATTTTGAGCCTAGGCTTGACTACTTACGATCGGAAGAGCGTCGTGTAGCCCATATAAGAAATTCGTGTTCTAGGCTTGACTACTTTAGATCGGAAGAGCGTCGTGTAGAGCGTCGTGTAGCCCATATAAGAAATTTTGAGCCTCAGCTTGACTTAGATCGGAAGAGCGTCGTGTCACCCATATAAGAAATTTTCTGAATAGGCTTGACTACTTTAGATCGGAAGAGCGTCGTGTAGCCTATATAAGAAACGGCGCGTCTAGGCTTGACTACTTTAGATCGGAAGAGCGTCAGCCCATATAAGAAATGCTCCGCCTAGGCTTGACTACTTTAGATCGGAAGAGCGTCGTGTAGCCCATATAAGAAATGCTCCGCCTAGGCTTGACTACTTTAGATCGGAAGAGCGTCGTGTAGCCCATATAAGAAATTTTCTGAATAGGCTTGACTACTTTAGATCGGAAGAGCGTCGTGTAGCCTATATAAGAAACGGCGCGTCTAGGCTTGACTACTTTAGATCGGAAGAGCGTCGTGTAGCTACACGACGCTCTTCCGATCTAAAGTAGTCAGACCTTAAAGGGAATATTTCTTATATGGGCTACACGACGCTCTTCCGATCTAAAAGTCGACTTACACGACGCTCTTCCGATCTAAAGTAGTCAAGCCTAGGAATAGTATTTCTTATATGGGCTACACAACGCTCTTCCGATCTAAAAGTAGACTACGACGCTCTTCCGATCTAAAGTAGTCAGACCCCATATAAGAAATTTCCCCTGTAGGCTTGACTACTTTAGATCGGAAGAGCGTCGTGTAGTCTACTTTTAGATCGGAAGAGCGTCGTGTAGCCCATATAAGAAATACTATTCCTAGGCTTGACTACTTTAGATCGGAAGAGCGTCGTGTAGTCTATTTTTAGATCGGAAGAGCGTCGTGTAGCCCATATAAGAAATATTCCCTTTAGGCTTGACTACTTTAGATCGGAAGAGCGTCGTGTAGTCTATTTCAGATCGGAAGAGCGTCGTGTAGCCCATATAAGAAATACTATTCCTAGGCTTGACTACTTTAGATCGGAAGAGCGTCGTGTAGA

**Supplementary Materials Figure S2 Example of TSO concatemerization sequence.**

Example of a read sequenced on Oxford Nanopore Technologies’ PromethION platform consisting of 26 repeats of TSO (highlighted in alternated shades of gray).

**(A)**


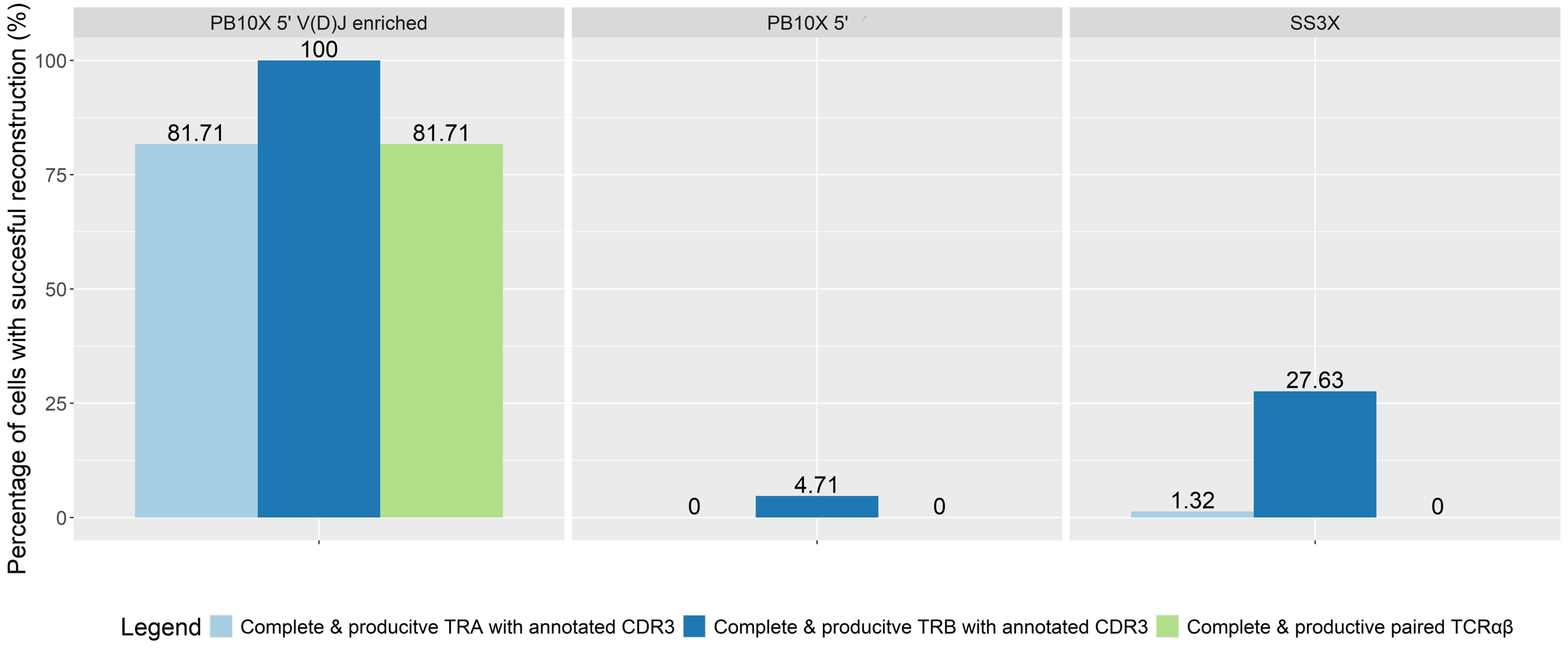


**(B)**


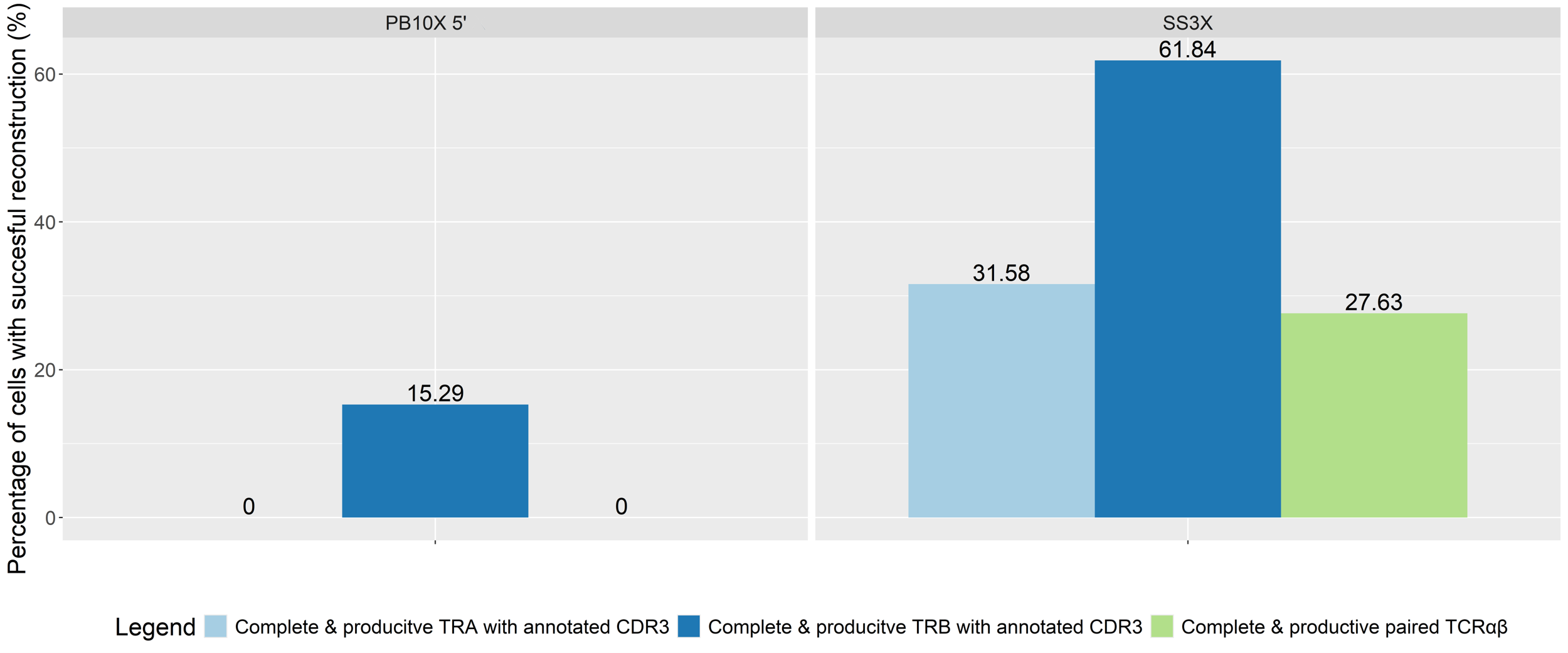


**(C)**


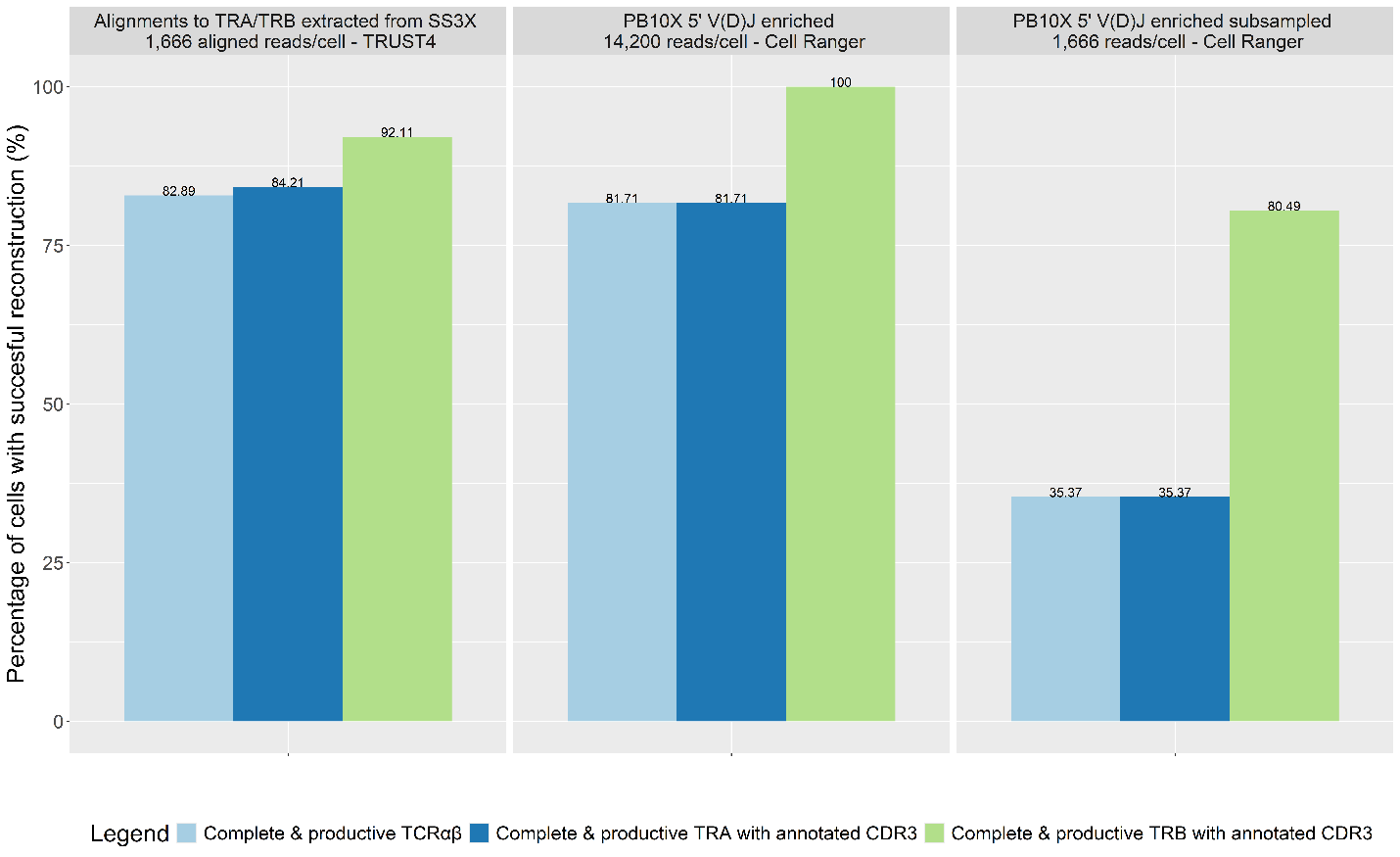


**Supplementary Materials Figure S3** **Percentages of cells with successful TCR profiling across the different strategies and subsampling settings.** The percentage of cells with successful profiling of a complete, productive TCR α-chain, TCR β-chain and TCR αβ-pair are displayed after subsampling to sequencing depth of **(A)** PB10X V(D)J-enriched (14,200 reads/cell), and **(B)** PB10X gene expression (66,700 reads/cell) dataset. In **(C)**, the PB10X V(D)J-enriched dataset was subsampled to an average of 1,666 reads/cell, i.e. the average number of reads per cell aligned to the *TRA* or *TRB* gene in the complete SS3X dataset. For analysis of TCR recovery rates of the PB10X-V(D)J-enriched strategy Cell Ranger was used, while for TCR sequence reconstructions based on SS3X and PB10X 5’ without specific V(D)J enrichment the TRUST4 algorithm was used.


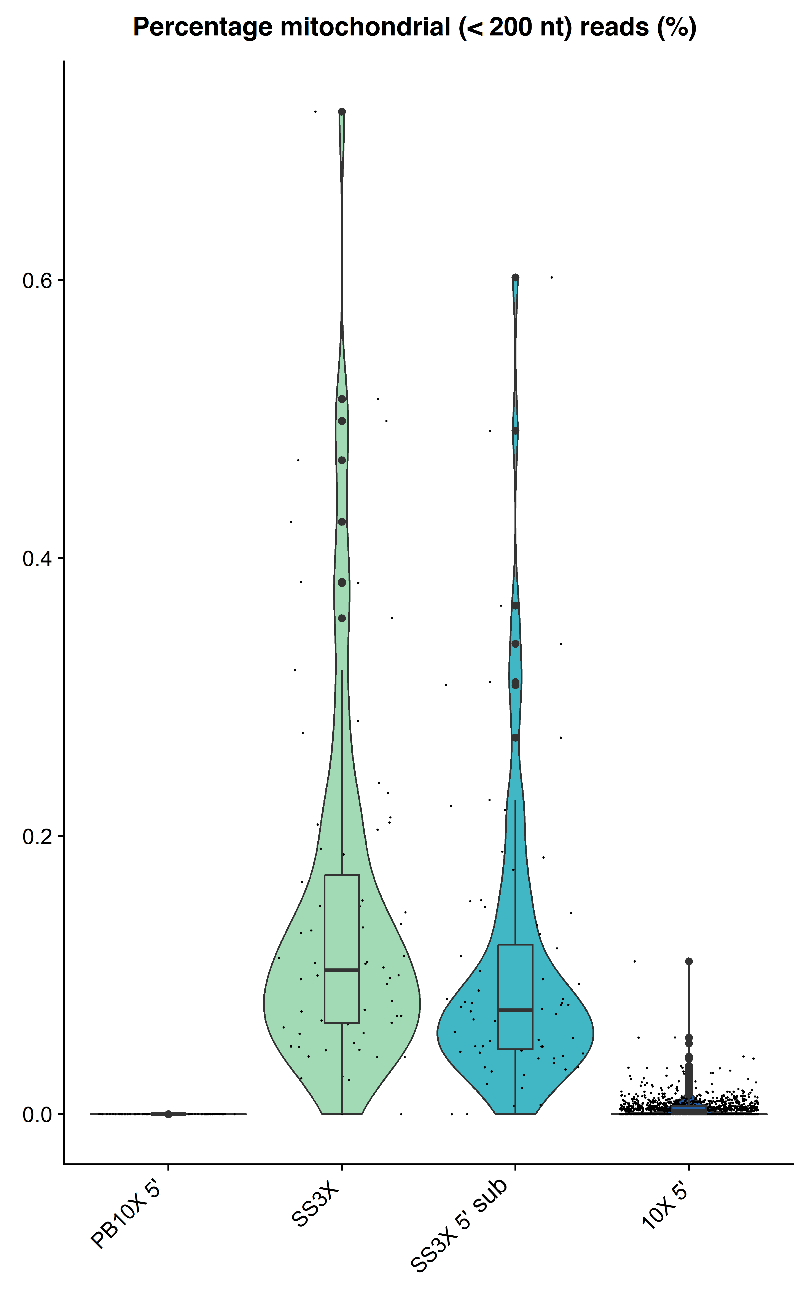


**Supplementary Materials Figure S4** Percentage of reads mapping to short mitochondrial genes (< 200 nt) per cell across the different strategies.

Violin plot showing the proportion of mitochondrial reads shorter than 200 nt, primarily mitochondrial tRNAs, in PB10X 5’, SS3X (full and subsampled), and 10X 5′ datasets. The lower fraction observed in PB10X 5’ may be attributed to the intermediate AMPure purification step, which preferentially removes shorter fragments.

|  | **96 cells** | **384 cells** |
| --- | --- | --- |
| **V(D)J only** |  |  |
| **GEX only** |  |  |
| **V(D)J + GEX** |  |  |

**Supplementary Materials Figure S5 Quantitative cost comparison of PB10X, SS3X and 5’ 10X Genomics workflows across different conditions.**

Total library preparation and sequencing costs (in EUR) for three workflows, PB10X, SS3X, and 5′ 10X Genomics, are shown for two low-throughput experimental scales (96 and 384 cells) and three library types: V(D)J only, gene expression (GEX) only, and combined V(D)J + GEX. For PB10X, three configurations of uniquely barcoded TSOs were evaluated (24, 96, and 384 TSOs), reflecting different pooling strategies. Cost calculations exclude cell isolation and consumables. PB10X consistently shows the lowest total cost across most configurations, if the number of barcoded TSOs is optimized for the number of cells processed.
